# CD155 Cooperates with PD-1/PD-L1 to Promote Proliferation of Esophageal Squamous Cancer Cells via PI3K/Akt and MAPK Signaling Pathways

**DOI:** 10.3390/cancers14225610

**Published:** 2022-11-15

**Authors:** Xiyang Tang, Jie Yang, Anping Shi, Yanlu Xiong, Miaomiao Wen, Zhonglin Luo, Huanhuan Tian, Kaifu Zheng, Yujian Liu, Chen Shu, Nan Ma, Rui Wang, Jinbo Zhao

**Affiliations:** 1 Department of Thoracic Surgery, Tangdu Hospital, Air Force Medical University, 569 Xinsi Road, Xi’an 710038, China; 2 Department of Radiology, Functional and Molecular Imaging Key Lab of Shaanxi Province, Tangdu Hospital, Fourth Military Medical University (Air Force Medical University), 569 Xinsi Road, Xi’an 710038, China; 3 Department of Cardiothoracic Surgery, Peace Hospital, Changzhi Medical College, 161 Jiefang East Street, Changzhi 046000, China; 4 Department of Ophthalmology, Tangdu Hospital, Air Force Medical University, 569 Xinsi Road, Xi’an 710038, China; 5 Medical Department, Tangdu Hospital, Air Force Medical University, 569 Xinsi Road, Xi’an 710038, China

**Keywords:** CD155, PD-1/PD-L1, immunotherapy, esophageal squamous cell cancer

## Abstract

**Simple Summary:**

Some immunotherapies, such as anti-PD1 and anti-PD-L1 treatments, have been used to treat various tumors. However, they are less efficient against esophageal cancer, partially owing to a lack of research on the cellular and molecular mechanisms of this cancer. Therefore, various emerging immune checkpoints have been discovered in this post-PD-1 era. One such immune checkpoint is CD155, a protein belonging to the Nectin-like family and expressed on the surface of cancer and immune cells. Exploring the mechanisms and therapeutic applications of these immune checkpoints may effectively improve cellular responses to immunotherapies. In this study, we aimed to explore the role of CD155 in esophageal squamous cell cancer (ESCA) and its underlying molecular mechanism. CD155 was positively associated with PD-1/PD-L1 expression and could support ESCA proliferation. The downregulation of CD155 expression inhibited ESCA cell proliferation by impairing the cell cycle and inducing cell apoptosis. This occurred via the inhibition of PI3K/Akt and MAPK signaling pathways. In addition, Nectin3 may be the ligand of CD155 and may be involved in ESCA proliferation. Thus, our study suggests novel targets for tumor therapy, especially for ESCA treatment.

**Abstract:**

Background: Esophageal cancer is still a leading cause of death among all tumors in males, with unsatisfactory responses to novel immunotherapies such as anti-PD-1 agents. Herein, we explored the role of CD155 in esophageal squamous cell cancer (ESCA) and its underlying molecular mechanisms. Methods: Publicly available datasets were used for differential gene expression and immune infiltration analyses, and their correlation with patient survival. A total of 322 ESCA and 161 paracancer samples were collected and evaluated by performing immunohistochemistry and the H score was obtained by performing semiquantitative analysis. In vitro transfection of ESCA cell lines with lentivirus vectors targeting CD155 was performed to knockdown the protein. These cells were analyzed by conducting RNA sequencing, and the effects of CD155 knockdown on cell cycle and apoptosis were verified with flow cytometry and Western blotting. In addition, in vivo experiments using these engineered cell lines were performed to determine the role of CD155 in tumor formation. A small interfering RNA-mediated knockdown of Nectin3 was used to determine whether it phenocopied the profile of CD155 knockdown. Results: CD155 is highly expressed in ESCA tissues and is positively associated with *PD1*, *PDL1*, *CD4*, *IL2RA*, and *S100A9* expression. Furthermore, CD155 knockdown inhibited ESCA cells’ proliferation by impairing the cell cycle and inducing cell apoptosis. Bioinformatics analysis of the gene expression profile of these engineered cells showed that CD155 mainly contributed to the regulation of PI3K/Akt and MAPK signals. The downregulation of Nectin3 expression phenocopied the profile of CD155 knockdown. Discussion: CD155 may cooperate with PD-1/PD-L1 to support ESCA proliferation in ways other than regulating its underlying immune mechanisms. Indeed, CD155 downregulation can impair ESCA cell pro-cancerous behavior via the inhibition of the PI3K/Akt and MAPK signaling pathways. Moreover, Nectin3 may be a ligand of CD155 and participate in the regulation of ESCA cells’ proliferation. Hence, the inhibition of CD155 may enhance the therapeutic effect of anti-PD-1 immunotherapies in ESCA.

## 1. Introduction

Esophageal cancer is the sixth leading cause of death among all tumors [1]. In particular, China has the largest number of patients with esophageal cancer worldwide, with about 193,000 deaths per year, of which 90% are due to esophageal squamous cell cancer (ESCA) [2,3]. Currently, immunotherapy is the most widely adopted anticancer treatment option, especially for lung cancer, melanoma, and hepatocellular carcinoma [4,5,6]. Nevertheless, the immune checkpoint therapy anti-programmed death protein 1 (PD-1) has failed to induce a satisfactory therapeutic effect in advanced and refractory esophageal cancer. Indeed, the overall response rate in refractory esophageal cancer reaches only 8% [7]; however, more manageable toxicity occurs in advanced patients [8]. Thus, the application of immunotherapies, such as using anti-PD-1 agents, in esophageal cancer warrants further research.

PD-1 is expressed on the surface of multiple immune cells, including macrophages and B, T, natural killer, and natural killer T cells [9], whereas its ligand PD-L1 is mainly expressed by cancer cells, such as gastric, non-small cell lung, and breast cancer cells [10]. The interaction between PD-1 and PD-L1 promotes strong inhibitory immune signals in the tumor microenvironment, in particular toward T cell inhibition [11]. Moreover, both PD-1 and PD-L1 were shown to promote tumor proliferation by regulating the cell cycle of several cancer cell types [12,13,14,15,16], including esophageal cancer cells [17], further demonstrating the pro-proliferative role of PD-1/PD-L1 signals in cancer.

A preliminary study of the differential expression of 35 immune checkpoint genes in ESCA using publicly available data suggested that CD155 could mediate important regulatory effects in this cancer type (Figure S1). CD155 (also named poliovirus receptor or PVR) is an immune checkpoint protein that belongs to the Nectin-like family and is expressed on the surface of cancer and immune cells [18,19]. CD155 mainly regulates the immune activity of natural killer [20,21] and T [22] cells in the tumor microenvironment of various cancers. Nectin3 (also called PVRL3) was proven to be one of the ligands of CD155 and may bind with CD155 in regulating cell movement and adhesion [23,24]. Based on the unsatisfactory therapeutic effect of anti-PD-1 therapies in ESCA, CD155 may represent a good therapeutic candidate to achieve improved anticancer responses with fewer immune-related adverse events. Therefore, herein, we explored the role of CD155 in ESCA for the first time, including its relationship with PD-1/PD-L1 signals in the underlying mechanisms of this life-threatening disease.

## 2. Materials and Methods

### 2.1. Analysis of Gene Expression and Immune Infiltration

Data from the Gene Expression Profiling Interactive Analysis database (GEPIA, http://gepia.cancer-pku.cn/, accessed on 4 July 2022), which comprise RNA-sequencing data from the Genotypic-Tissue Expression project and The Cancer Genome Atlas, were used for gene expression and gene–gene correlation analyses [25]. Pancancer analysis was performed using the University of Alabama at Birmingham Cancer data analysis platform (UALCAN, http://ualcan.path.uab.edu/, accessed on 4 July 2022) [26] and the Assistant for Clinical Bioinformatics database (ACLBI, www.aclbi.com, accessed on 4 July 2022), which also facilitated simultaneous immune infiltration analysis. The relationship between gene expression profiles, tumor immune infiltration, and patient survival was evaluated using data from the GEPIA and UALCAN databases.

### 2.2. Analysis of Gene and Protein Interaction

Data from the GeneMANIA (https://genemania.org/, accessed on 7 July 2022) database were used for gene prioritization network integration and gene–gene interaction prediction [27], and protein–protein interaction prediction was performed in the STRING database (www.string-db.org, accessed on 8 July 2022) [28].

### 2.3. ESCA Sample Collection

A total of 322 ESCA and 161 paracancer samples were collected from patients who were surgically treated in Tangdu Hospital (Xi’an, China). The inclusion criteria and exclusion criteria were formulated as previously reported [29]. This study was approved by the Ethics Committee of the Air Force Medical University (No. 202108-05).

### 2.4. Immunohistochemistry

All tissue samples were embedded in paraffin and cut into 3 μm slices. Immunohistochemistry was conducted as previously reported [29]. The tissue slices were incubated overnight at 4 °C with anti-CD155, anti-PD1, anti-PD-L1, anti-CD4, anti-IL-2RA, anti-S100A9, anti-CD3D, anti-CD8, anti-FOXP3, anti-TPSB2, anti-CD79A, anti-GNLY, and anti-Nectin3 (Signalway Antibody, Greenbelt, MD, USA), respectively.

A semiquantitative analysis of the H score was performed using the Aipathwell.v2 software as follows:

H score = ∑(pi × i) = (percentage of weak intensity × 1) + (percentage of moderate intensity × 2) + (percentage of strong intensity × 3) [30,31,32].

To assess the correlation between CD155 and PD-1/PD-L1, H scores of <25% were considered as negative expression. The semiquantitative analysis was supported by Wuhan Servicebio Technology Co. (Wuhan, China).

### 2.5. Immunofluorescence Analysis

TE1 and KYSE-520 (K520) human esophageal squamous cancer cells were cultured in 6-well plates until they reached 70% density. A membrane breaking solution (100 μL; Servicebio) was added to the wells and incubated at 25 °C for 20 min. The cells were then washed three times with PBS (phosphate-buffered saline solution) and were incubated overnight at 4 °C with anti-CD155 polyclonal antibody (1:50 dilution; Signalway Antibody, Greenbelt, MD, USA); the respective secondary antibody was incubated at 25 °C for 50 min. To stain the nucleus of the cells, 4′,6-diamidino-2-phenylindole (DAPI) was used.

### 2.6. Cell Culture

The cultures of TE1 and K520 human esophageal squamous cancer cells (iCell Bioscience Inc., Shanghai, China) were the same as previously reported [29].

### 2.7. CD155 and Nectin3 Knockdown

CD155 was knocked down (KD) in TE1 and K520 cells using a lentiviral vector synthesized by CytoBiotech (Guangzhou, China) that harbored a luciferase tag and a short hairpin RNA which specifically targets *CD155* through the sequence 5′–CTGTGAACCTCACCGTGTA–3′. Nectin3 was knocked down in TE1 and K520 cells using siRNA (RiboBio, Guangzhou, China). The sequence of the siRNA used was 5′-GACATCCGATACTCTTTCA-3′. A non-targeting vector was used as the control (NC).

### 2.8. Xenograft Tumor Experiment

Male mice (BALB/cJGpt-Foxn1nu/Gpt; 5 weeks old; 20–25 g) were purchased from GemPharmatech (Beijing, China). The mice were randomly divided into two groups (*n* = 5 in each group) and injected subcutaneously in the back with CD155_NC or CD155_KD TE1 cells (1 × 10^6^ cells/animal). Within 7–17 days post-injection, the volume of the tumors was measured daily based on the two largest perpendicular dimensions. The tumor volume (mm^3^) was calculated as (tumor length [mm] × square of tumor width [mm]^2^)/2. All in vivo experiments were reviewed and approved by the Ethics Committee of the Air Force Medical University (No. 202203-145).

### 2.9. Flow Cytometry

For apoptosis analysis, the cells were washed once with phosphate-buffered saline solution and resuspended in a 100 μL (1×) binding buffer. Fluorochrome-conjugated Annexin V (5 µL) was added and incubated for 10–15 min, protected from light at room temperature, and immediately analyzed using a Beckman Coulter (Brea, CA, USA) flow cytometer.

### 2.10. Western Blotting

Western blotting was conducted as previously reported [29]. The membranes were incubated overnight at 4 °C with the following specific antibodies: anti-β-actin, anti-CD155, anti-PI3K, anti-phosphorylated Akt (Ser473), anti-P38, anti-P38 MAPK, anti-ERK1/2, anti-phosphorylated ERK1/2 (Thr202/Tyr204), anti-JNK1/2/3, anti-phosphorylated JNK1/2/3 (Thr183/Tyr185), anti-cyclin A2, anti-cyclin B1, anti-cyclin D1, anti-cyclin E1, anti-CDK2, anti-CDK4, anti-CDK6, anti-caspase 3, anti-caspase 7, anti-caspase 9, anti-cleaved caspase 9, anti-PARP 1, anti-cleaved PARP, and anti-Nectin3 (Signalway Antibody, Greenbelt, MD, USA), respectively.

### 2.11. Cell Proliferation Analysis

CD155_NC and CD155_KD ESCA cells were collected; the analyses in a real-time cell analyzer (Agilent Technologies, Santa Clara, CA, USA) and cell proliferation were conducted as previously reported [29]. Similarly, 1000 cells from each of these two groups were seeded onto 6-well plates, cultured for the next 10 days, and washed three times with phosphate-buffered saline solution; then, all of the cells were fixed in methanol for 25 min and stained with 5% crystal violet for 40 min.

### 2.12. mRNA Sequencing and Analysis

RNA from CD155_NC and CD155_KD TE1 cells was isolated using TRIzol Reagent (Thermo Fisher Scientific, Carlsbad, USA) and the samples were submitted to Genergy Bio-Technology Co. (Shanghai, China) for mRNA sequencing and analysis.

### 2.13. Statistical Analysis

Prism 8.2.1 (GraphPad Software, San Diego, CA, USA) and SPSS 26 (IBM Corp, Armonk, NY, USA) were used for statistical analysis. Statistical data are expressed as mean ± standard deviation. Differences between the two groups were evaluated using the Student’s *t* test. H scores without equal standard deviations were analyzed using the Mann–Whitney test for rank comparison. *p* ≤ 0.05 was considered statistically significant.

## 3. Results

### 3.1. CD155 Is Highly Expressed in ESCA and Is Associated with Poor Patient Prognosis

**Figure 1:**
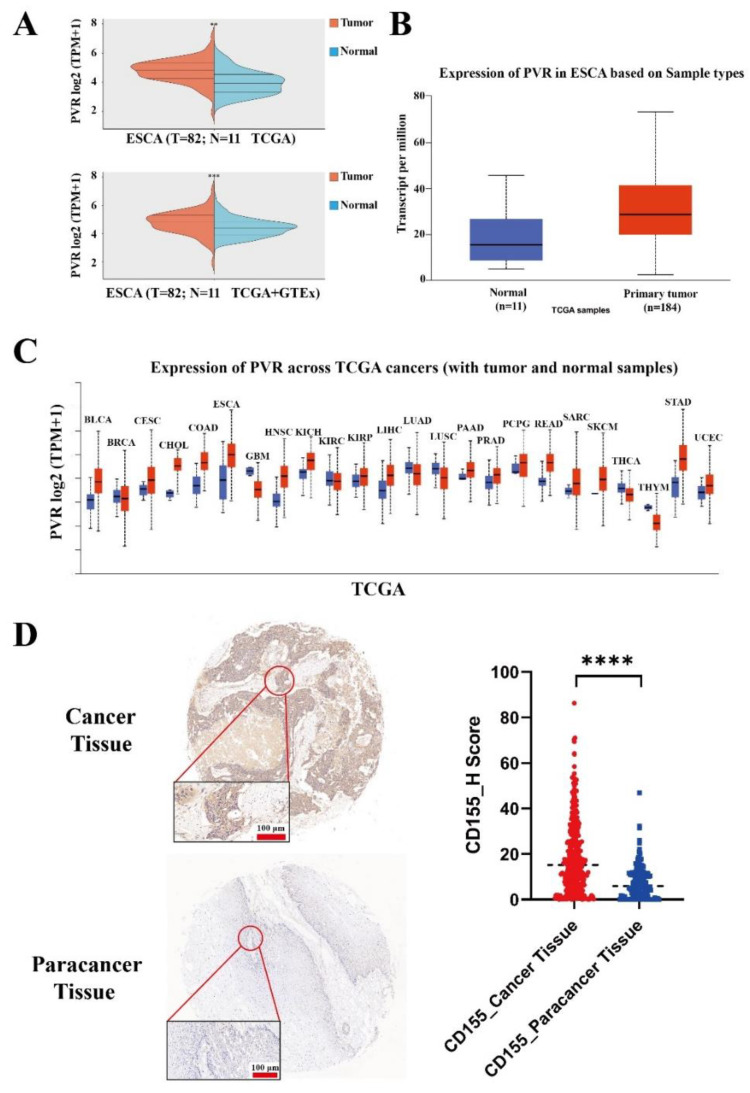
CD155 is highly expressed in ESCA. Data from (**A**) ACLBI and (**B**) UALCAN databases all showed higher expression of CD155 in ESCA compared to normal samples. Pancancer analysis from the (**C**) UALCAN databases showed higher CD155 expression could also be found in CHOL, COAD, and PAAD, as well as other tumors. (**D**) Further validation for the protein level of CD155 was evaluated by immunohistochemistry. ESCA: esophageal squamous cancer; CHOL: cholangio carcinoma; COAD: colon adenocarcinoma; PAAD: pancreatic adenocarcinoma. ** *p* < 0.01, *** *p* < 0.001, **** *p* < 0.0001.

The analysis of publicly available data in different databases showed that CD155 was highly expressed in ESCA (Figure 1A,B). In agreement with this finding, a pancancer analysis confirmed the high expression of CD155 in ESCA, as well as in other cancers, including cholangiocarcinoma, colon adenocarcinoma, and pancreatic adenocarcinoma (Figure 1C) (Supplementary Figure S2). To further validate these results, 322 primary ESCA and 161 paracancerous tissues were evaluated by performing immunohistochemistry, which revealed that CD155 was significantly more expressed in ESCA tissues than in healthy cells (*p* < 0.0001) (Figure 1D). Altogether, these results demonstrate that CD155 is highly expressed in ESCA, thus potentially playing an important role in ESCA development.

**Figure 2:**
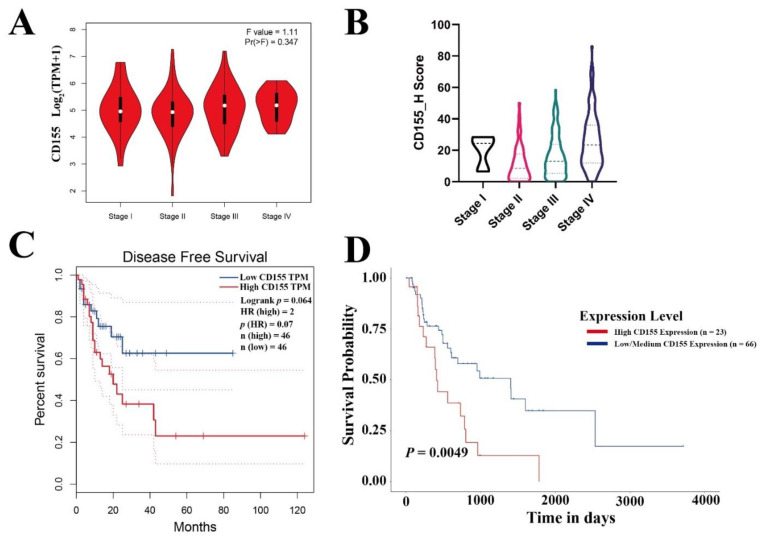
CD155 is expressed in all cancer stages and associated with poor patient prognosis. (**A**) The expression of CD155 in the different stages of ESCA was evaluated using the GEPIA database, the numbers in the Y axis indicate the transcription level of CD155. (**B**) The expression of CD155 in the different stages of ESCA was validated by performing immunohistochemistry, the numbers in the Y axis indicate the protein level of CD155. Survival analyses related to CD155 were performed in the (**C**) GEPIA and (**D**) UALCAN databases.

CD155 expression was also evaluated concerning the different stages of ESCA. Noteworthily, CD155 was expressed in all cancer stages (Figure 2A,B). Furthermore, the patient survival analysis showed that a high expression of CD155 predicted poor ESCA prognosis (Figure 2C,D).

### 3.2. CD155 Is Positively Associated with the Expression of CD4, IL-2Rα and S100A9 in ESCA

The gene–gene interaction analysis revealed that *CD155* may interact with *CD96*, *CD226*, and *NECTIN3* via physical interactions (Figure S3A). In addition, the protein–protein interaction analysis further confirmed that CD155 can interact with CD96 and CD226 (Figure S3B).

**Figure 3:**
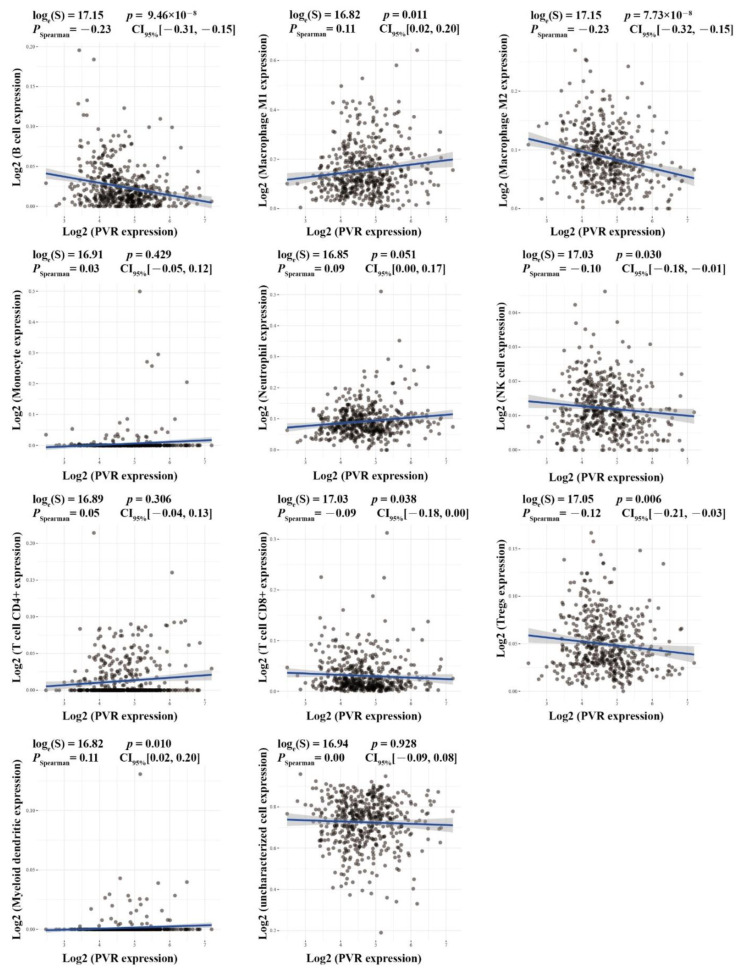
CD155 can be involved in immune infiltration. Immune infiltration analysis was performed in the ACLBI databases.

**Figure 4:**
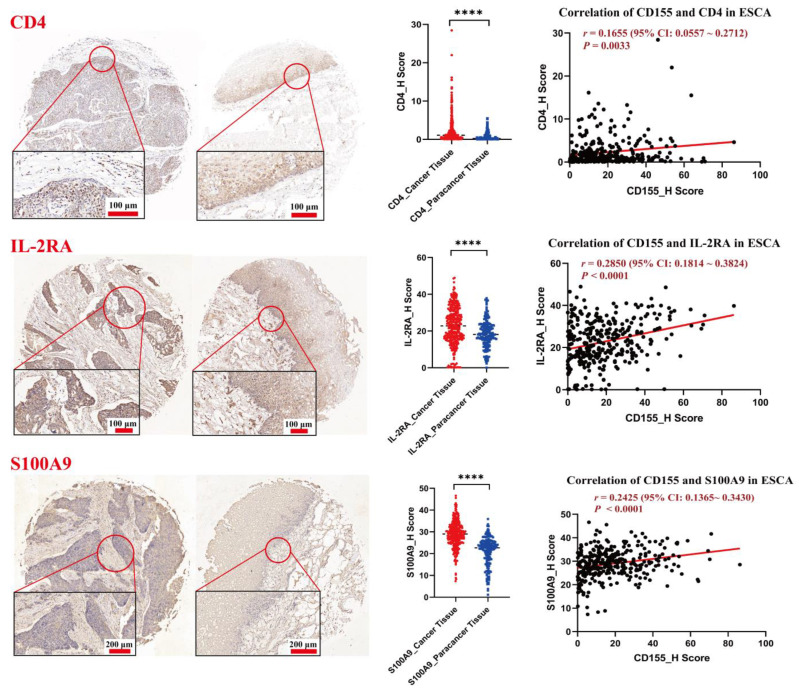
CD155 is positively associated with the expression of CD4, IL-2RA, and S100A9. Three immune-related markers were stained by performing immunohistochemistry. A correlation analysis between CD155 and immune markers was performed, and positive correlations were observed between CD155 and CD4 (*r* = 0.1655, *p* = 0.0033), IL-2RA (*r* = 0.2850, *p* < 0.0001), and S100A9 (*r* = 0.2425, *p* < 0.0001). **** *p* < 0.0001.

To further explore the potential role of CD155 in ESCA, an immune infiltration analysis was performed. Interestingly, *CD155* expression was found to be positively associated with the levels of M1 macrophages (*r* = 0.11, *p* = 0.011), myeloid dendritic cells (*r* = 0.11, *p* = 0.01), and neutrophils (*r* = 0.09, *p* = 0.051), but negatively associated with the levels of B cells (*r* = −0.23, *p* = 9.46 × 10^−8^), M2 macrophages (*r* = −0.23, *p* = 7.73 × 10^−8^), natural killer cells (*r* = −0.10, *p* = 0.03), CD8^+^ T cells (*r* = −0.09, *p* = 0.038), and T regulatory cells (*r* = −0.12, *p* = 0.006) (Figure 3). A further protein analysis confirmed that both CD8 and CD79A were decreased in ESCA tissues compared with the controls (*p* < 0.05), as well as TPSB2 (*p* < 0.01); however, the expressions of interleukin (IL)-2 receptor α (IL-2Rα) and S100A9 were all significantly elevated in ESCA (*p* < 0.0001). These results were confirmed by performing a correlation analysis, which showed significantly positive correlations between *CD155* and *CD4* (CD4^+^ T cell marker, *r* = 0.1655, *p* = 0.0033), and *IL2RA* (B cell marker, *r* = 0.2850, *p* < 0.0001) and *S100A9* (neutrophil marker, *r* = 0.2425, *p* < 0.0001) (Figure 4); however, there was no correlation with other immune markers (Figure S4). Thus, CD155 may contribute to regulating immune infiltration in ESCA, especially the regulation of CD4^+^ T cells, B cells, and neutrophils.

### 3.3. CD155 Can Cooperate with PD-1/PD-L1

**Figure 5:**
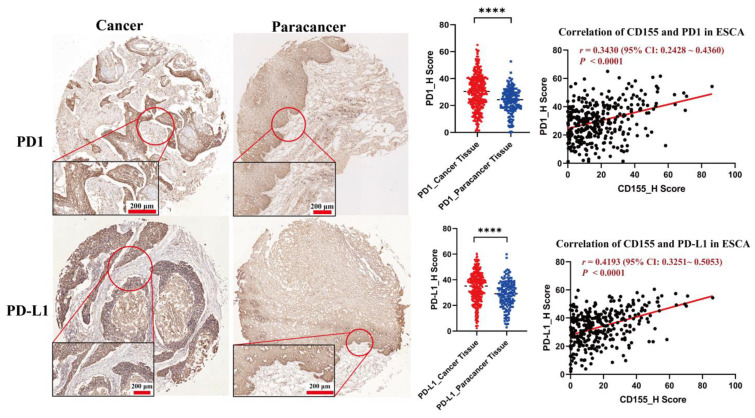
[CD155 is positively associated with PD1/PD-L1]. The correlation analysis between CD155 and PD1 or PD-L1 was performed, and CD155 was positively associated with PD1 (*r* = 0.343, *p* < 0.0001) and PD-L1 (*r* = 0.4193, *p* < 0.0001). A total of 322 ESCA samples was divided into 8 categories based on the expression type of CD155, PD1, and PD-L1; triple positive samples accounted for 58.1%, and double positive samples, PD1^+^CD155^+^, or PD-L1^+^CD155^+^ accounted for 10.9%. **** *p* < 0.0001.

**Figure 6:**
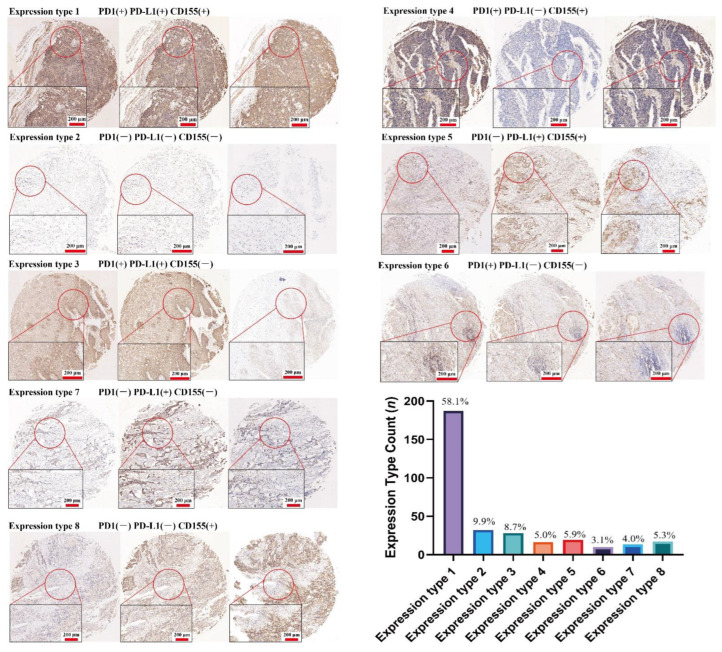
CD155 is tightly associated with PD1/PD-L1. A total of 322 ESCA samples were divided into 8 categories based on the expression type of CD155, PD1, and PD-L1; triple positive samples accounted for 58.1%, and double positive samples, PD1^+^CD155^+^, or PD-L1^+^CD155^+^ accounted for 10.9%.

To further analyze the relationship between CD155 and PD-1/PD-L1, 322 ESCA samples were divided into eight categories according to their phenotype: PD-1^+^PD-L1^+^CD155^+^, PD-1^−^PD-L1^−^CD155^−^, PD-1^+^PD-L1^+^CD155^−^, PD-1^+^PD-L1^−^CD155^+^, and PD-1^−^PD-L1^+^CD155^+^, PD-1^+^PD-L1^−^ CD155^−^, PD-1^−^PD-L1^+^CD155^−^, and PD-1^−^PD-L1^−^CD155^+^. For this classification, quartiles were considered as cutoff values and H scores of less than 25% were considered as negative expressions. Among all samples, triple and double positive samples (PD-1^+^CD155^+^ or PD-L1^+^CD155^+^) accounted for 58.1% and 10.9%, respectively, further suggesting that CD155 is highly associated with PD1 and PD-L1 expression (Figure 5 and Figure 6). Hence, CD155 may cooperate with PD1/PD-L1 and may have an impact on the therapeutic effect of anti-PD1/PD-L1 treatments in ESCA.

### 3.4. CD155 Can Regulate the PI3K/Akt and MAPK Pathways in ESCA

**Figure 7:**
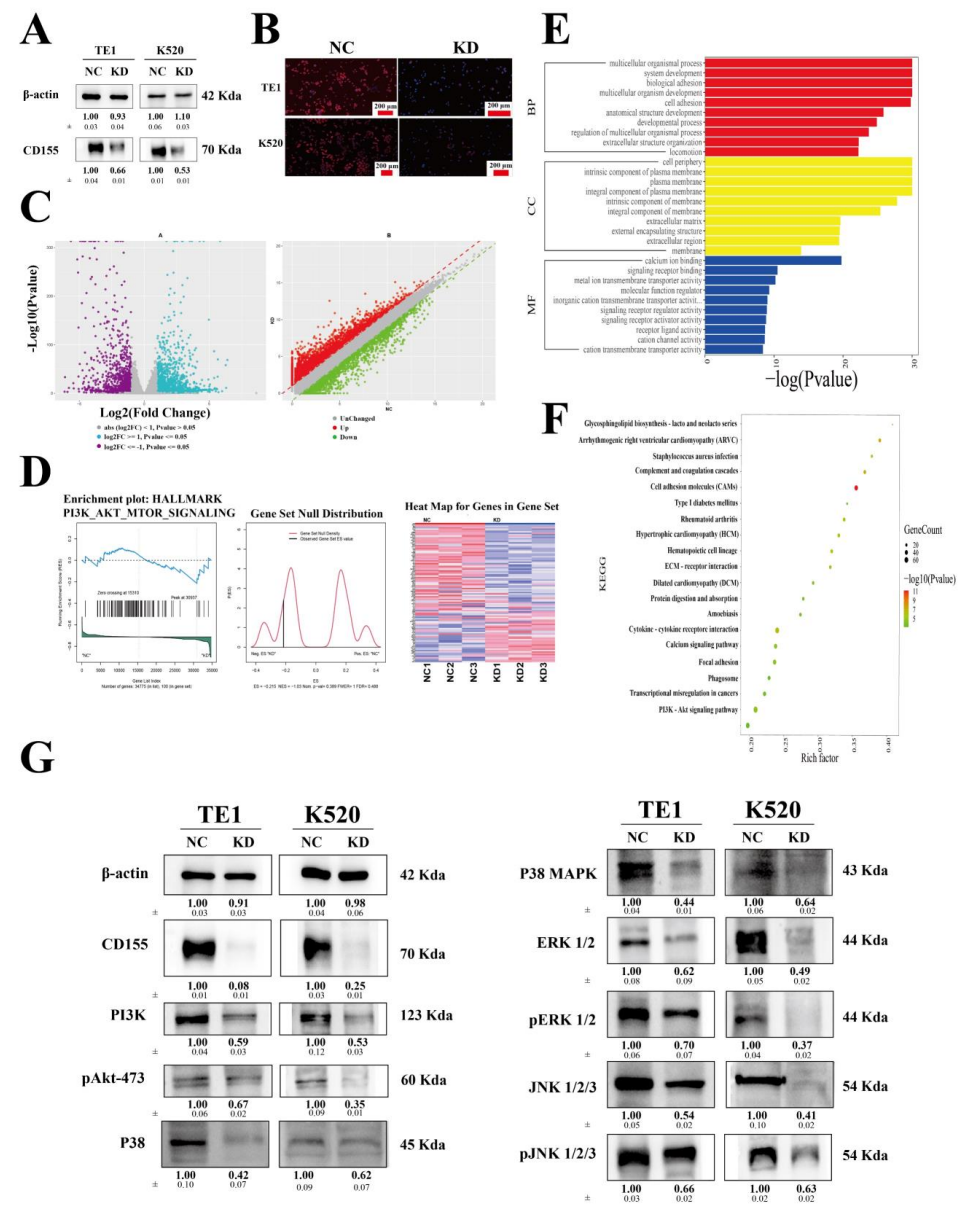
CD155 can regulate the PI3K/Akt and MAPK pathways in ESCA. (**A**) Western blot and (**B**) immunofluorescence data confirmed the decreased expression of CD155 in ESCA cells. (**C**) RNA sequencing of these cells and a respective (**D**) GSEA analyses were conducted, followed by (**E**) GO and (**F**) KEGG. KEGG and GSEA analyses both indicated that the CD155-related differentially expressed genes were mainly associated with the PI3K/Akt and MAPK signaling pathways. (**G**) The two signaling pathways related to CD155 were verified with Western blotting.The original WB Blots of subfigure (**A**) is Figure S5. The original WB Blots of subfigure (**G**) are Figure S6.

To further explore the potential cellular and molecular effects of CD155 in ESCA, we analyzed TE1 and K520 ESCA cells that were genetically engineered to lack CD155 expression. Western blot and immunofluorescence data confirmed the decreased expression of CD155 in ESCA cells, especially in the cytoplasm (Figure 7A,B). The RNA sequencing of these cells and respective gene ontology analyses showed that CD155 mainly contributed to developmental processes and signaling receptor binding, which could be associated with tumor growth. In addition, KEGG (Kyoto Encyclopedia of Genes and Genomes) and GSEA (Gene Set Enrichment Analyses) indicated that the CD155-related differentially expressed genes were mainly associated with the PI3K/Akt and MAPK signaling pathways (Figure 7C–F). To verify the signaling pathways that CD155 was involved with, the levels of major signaling proteins were evaluated. Interestingly, all the analyzed markers were decreased in the cells lacking CD155 (Figure 7G), further supporting that CD155 exerts an effect in ESCA cells via the PI3K/Akt and MAPK pathways.

### 3.5. CD155 Downregulation Inhibits ESCA Cell Proliferation by Impairing Cell Cycle and Inducing Cell Apoptosis

**Figure 8:**
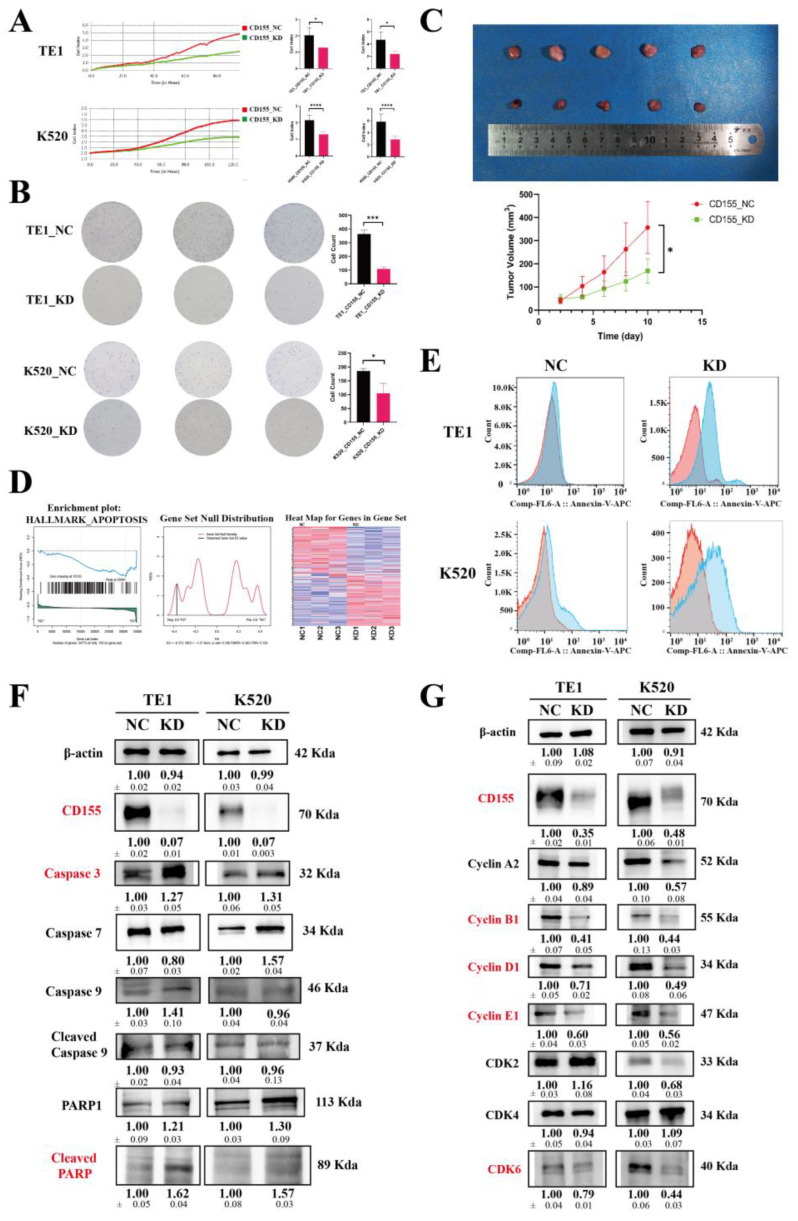
The downregulation of CD155 inhibits ESCA cells’ proliferation by inducing cell cycle S phase arrest and cell apoptosis. (**A**) Real-time cell analyzer, (**B**) colony formation assays, and (**C**) in vivo experiments were performed to validate the proliferation between CD155_NC and CD155_KD groups in TE1 cells and K520 cells. These cells showed increased levels of cell apoptosis, supported by (**D**) GSEA analysis from RNA-seq (**E**) cell apoptosis flow analysis, and (**F**) Western blotting revealed a higher level of caspase 3 and cleaved PARP in the CD155_KD group of TE1 and K520 cells. Moreover, (**G**) Western blot indicated that the downregulation of CD155 may induce a decreased level of Cyclin B1, D1, E1, and CDK6 in TE1 and K520 cells. * *p* < 0.05; *** *p* < 0.001; **** *p* < 0.0001.The original WB Blots of subfigure (**F**) is Figure S7. The original WB Blots of subfigure (**G**) are Figure S8.

Since the gene ontology results suggested that CD155 could be involved in cell developmental processes, we next explored the potential role of CD155 in cell cycle and apoptosis. The proliferation of TE1 and K520 ESCA cells was significantly inhibited for the CD155_KD group compared with the CD155_NC group, as determined using a real-time cell analyzer, colony formation assays, and in vivo experiments (Figure 8A–C). Moreover, these cells showed increased levels of cell apoptosis (Figure 8D,E), with elevated caspase 3 and cleaved PARP (Figure 8F). Noteworthily, ESCA cells lacking CD155 had an impaired cell cycle, with decreased levels of cyclins B1, D1, E1, and CDK6 (Figure 8G). In summary, CD155 downregulation can effectively inhibit the proliferation of ESCA cells by preventing their proliferation and promoting cell apoptosis.

### 3.6. CD155 May Interact with Nectin3 and Regulate ESCA Proliferation

**Figure 9:**
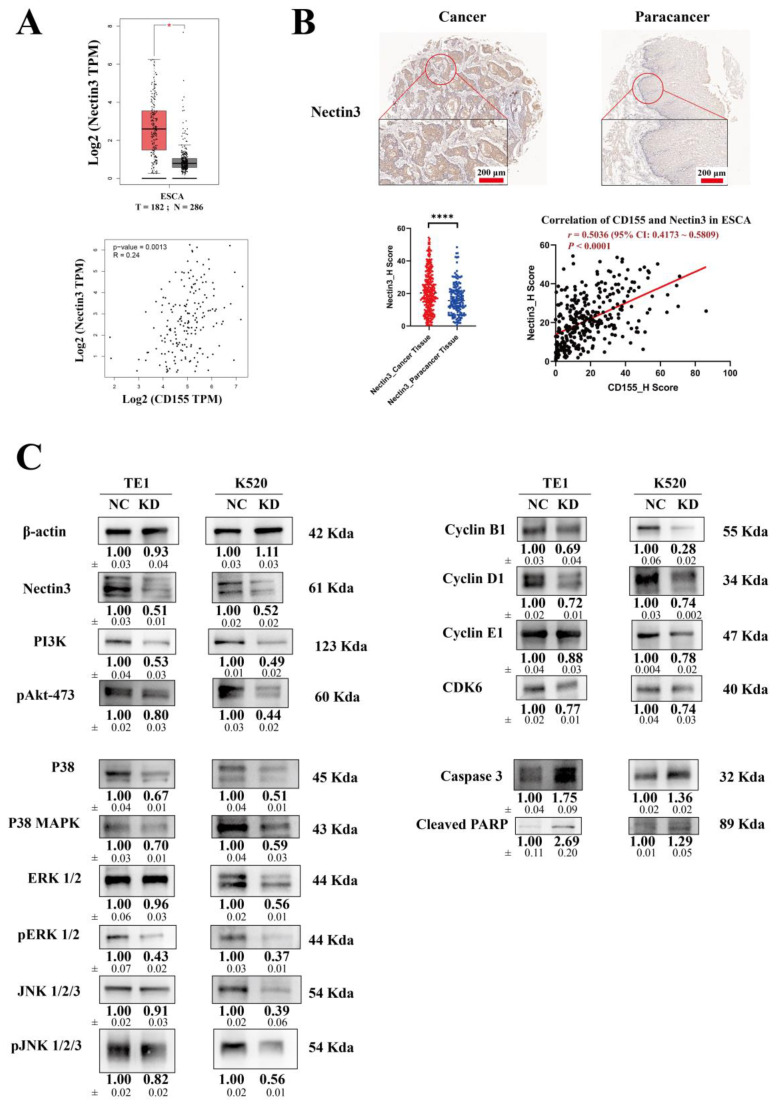
Downregulation of Nectin3 expression phenocopies the profile of CD155 knockdown. (**A**) Nectin3 expression and the correlation analysis between CD155 and Nectin3 expression were predicted using the GEPIA database and (**B**) validated at the protein level through tissue microarray. (**C**) Nectin3 is knocked down using siRNA. Changes similar to those of CD155 knockdown were seen in TE1 and K520 cells. * *p* < 0.05; **** *p* < 0.0001. The original WB Blots of subfigure (**C**) are Figure S9.

The knockdown of Nectin3 using siRNA phenocopies the profile of CD155 knockdown. Nectin3 was highly expressed in ESCA tissues compared to the adjacent tissues. In addition, it was positively associated with the expression of CD155, which was predicted in the GEPIA analysis (Figure 9A) and validated through tissue microarray (Figure 9B). Nectin3 was knocked down by siRNA, and its protein level was confirmed through Western blot analysis. The levels of PI3K, pAKT-473, P38, P38 MAPK, pERK1/2, pJNK1/2/3, Cyclin B1, Cyclin D1, and CDK6 were all decreased in TE1 and K520 cells. Furthermore, these cells showed increased levels of caspase 3 and cleaved PARP (Figure 9C). Similar results were obtained for the Western blot analysis of CD155 (Figure 8F,G), indicating that the knockdown of Nectin3 phenocopies the profile of CD155 knockdown. These results suggest that Nectin3 could be a ligand of CD155 and that their interaction could promote ESCA proliferation.

## 4. Discussion

Currently, the available immunotherapies lack efficiency in treating esophageal cancer [7,8], which can be, at least in part, explained by our lack of knowledge on its cellular and molecular mechanisms. Herein, we demonstrate that CD155 can be a valuable therapeutic candidate to treat these patients.

Overall, we found that CD155 is highly associated with PD-1/PD-L1 in ESCA. In particular, ESCA cells express high CD155 levels, which are positively associated with PD-1, PD-L1, CD4, IL-2Rα, and S100A9 levels, indicating that CD155 may exert immune regulatory effects in ESCA, especially toward CD4^+^ T cells, B cells, and neutrophils. Moreover, given the strong correlation between CD155 and PD1/PD-L1, CD155 may be related to the efficacy of anti-PD1/PD-L1 treatment in ESCA. However, we suggest that CD155 may cooperate with PD-1/PD-L1 in this cancer via a regulatory role other than immune regulation. In agreement with this hypothesis, PD-1 and PD-L1 were previously shown to promote tumor proliferation in multiple types of tumors, including esophageal cancer, with the inhibition of PD-1 or PD-L1 effectively preventing the proliferation and inducing the apoptosis of tumor cells [17]. Therefore, further studies on whether the co-expression of CD155 and PD-1/PD-L1 may further enhance tumor growth and progression, and their underlying regulatory mechanisms, are warranted.

In vitro and in vivo experimental assays showed that the downregulation of CD155 inhibits ESCA cells’ proliferation by preventing the expression of cell cycle-related proteins and by inducing apoptosis. Moreover, bioinformatics and experimental analyses of the expression profile of ESCA cells lacking CD155 suggested that CD155 downstream effects are mediated by the PI3K/Akt and MAPK signaling pathways, which are known to be involved in tumor proliferation [33,34,35]. Indeed, CD155 was previously shown to be able to directly bind to signaling proteins [36]. Thus, similar to PD-1, CD155 may play a positive regulatory role in the proliferation and progression of ESCA.

Herein, we determined that CD155 can interact with Nectin3 to promote ESCA proliferation. Nectin3 is a ligand of CD155. The interaction between these molecules can regulate cell behavior [24]. The protein level of Nectin3 is positively associated with CD155 expression in ESCA; both levels are higher in ESCA compared to those in adjacent tissues. Moreover, the downregulation of Nectin3 expression by siRNA phenocopies the profile of CD155 knockdown, especially with respect to the PI3K /Akt and MAPK signaling pathways, cell cycle-related proteins, and apoptosis-related proteins. Thus, we postulate that CD155 interacts with Nectin3 to promote ESCA proliferation. Similar results have been reported for multiple myeloma [36]. The binding of CD155 and Nectin3 can induce the adhesion of multiple myeloma cells to bone marrow stromal cells. Furthermore, CD155 can play multiple roles when binding with different ligands. When binding with the activating receptor DNAM-1, the cytotoxicity of NK and CD8+ T cells is promoted [37]. In contrast, binding with the inhibitory receptors TIGIT and CD96 can result in the inhibition of IFN-γ production and NK and T cell activity [21,38]. Similar immune regulation of CD155-mediated production can be observed in ESCA. However, further research is required.

In summary, the expression of CD155 is significantly positively associated with PD-1/PD-L1 signals, thereby regulating ESCA behavior by regulating its proliferation and viability via the PI3K/Akt and MAPK signaling pathways. Finding new strategies to block CD155, in addition to PD-1/PD-L1, may promote enhanced anticancer responses in patients with ESCA and help to achieve improved therapeutic outcomes.

## 5. Conclusions

In this study, we aimed to explore the role of CD155 in esophageal squamous cell cancer (ESCA) and its underlying molecular mechanism. CD155 was positively associated with PD-1/PD-L1 expression and could support ESCA proliferation. The downregulation of CD155 expression inhibited ESCA cell proliferation by impairing the cell cycle and inducing cell apoptosis. This occurred via the inhibition of PI3K/Akt and MAPK signaling pathways. In addition, Nectin3 may be the ligand of CD155 and may be involved in ESCA proliferation. Thus, our study suggests novel targets for tumor therapy, especially for ESCA treatment.

## Data Availability

The data that support the findings of this study are available from the corresponding author upon reasonable request. The RNA sequencing datasets generated in this study can be found in the data depository Figshare, and are publicly accessible at DOI: 10.6084/m9.figshare.20419200.

## Supplementary Materials

The following supporting information can be downloaded at: https://www.mdpi.com/article/10.3390/cancers14225610/s1, Figure S1: Differential expression of 35 immune checkpoint genes in ESCA; Figure S2: CD155 is also highly expressed in various tumors in addition to ESCA; Figure S3: The gene and protein interaction network of CD155; Figure S4: CD155 is not correlated with the expression of various immune markers; Figure S5: The original WB Blots of Figure 7A; Figure S6: The original WB Blots of Figure 7G; Figure S7: The original WB Blots of Figure 8F; Figure S8: The original WB Blots of Figure 8G; Figure S9: The original WB Blots of Figure 9C.

## Abbreviations

ESCA	Esophageal squamous cell cancer
IL	Interleukin
K520	KYSE-520
KD	Knockdown
NC	Non-targeting control
PD-1	Programmed death protein 1
PD-L1	Programmed death-ligand 1
