# Supplementary material for: CD155 Cooperates with PD-1/PD-L1 to Promote Proliferation of Esophageal Squamous Cancer Cells via PI3K/Akt and MAPK Signaling Pathways
Source: Cancers (Basel). 2022 Nov 15;14(22):5610. doi: 10.3390/cancers14225610 (PMC9688614; doi:10.3390/cancers14225610)
Supplement: Supplementary file 1 [file cancers-14-05610-s001.zip › cancers-1999480-supplementary.pdf]

Supplementary Materials

CD155 Cooperates with PD-1/PD-L1 to Promote Proliferation of Esophageal Squamous Cancer Cells via PI3K/Akt and MAPK Signaling Pathways

Xiyang Tang, Jie Yang, Anping Shi, Yanlu Xiong, Miaomiao Wen, Zhonglin Luo, Huanhuan Tian, Kaifu Zheng, Yujian Liu, Chen Shu, Nan Ma, Rui Wang and Jinbo Zhao

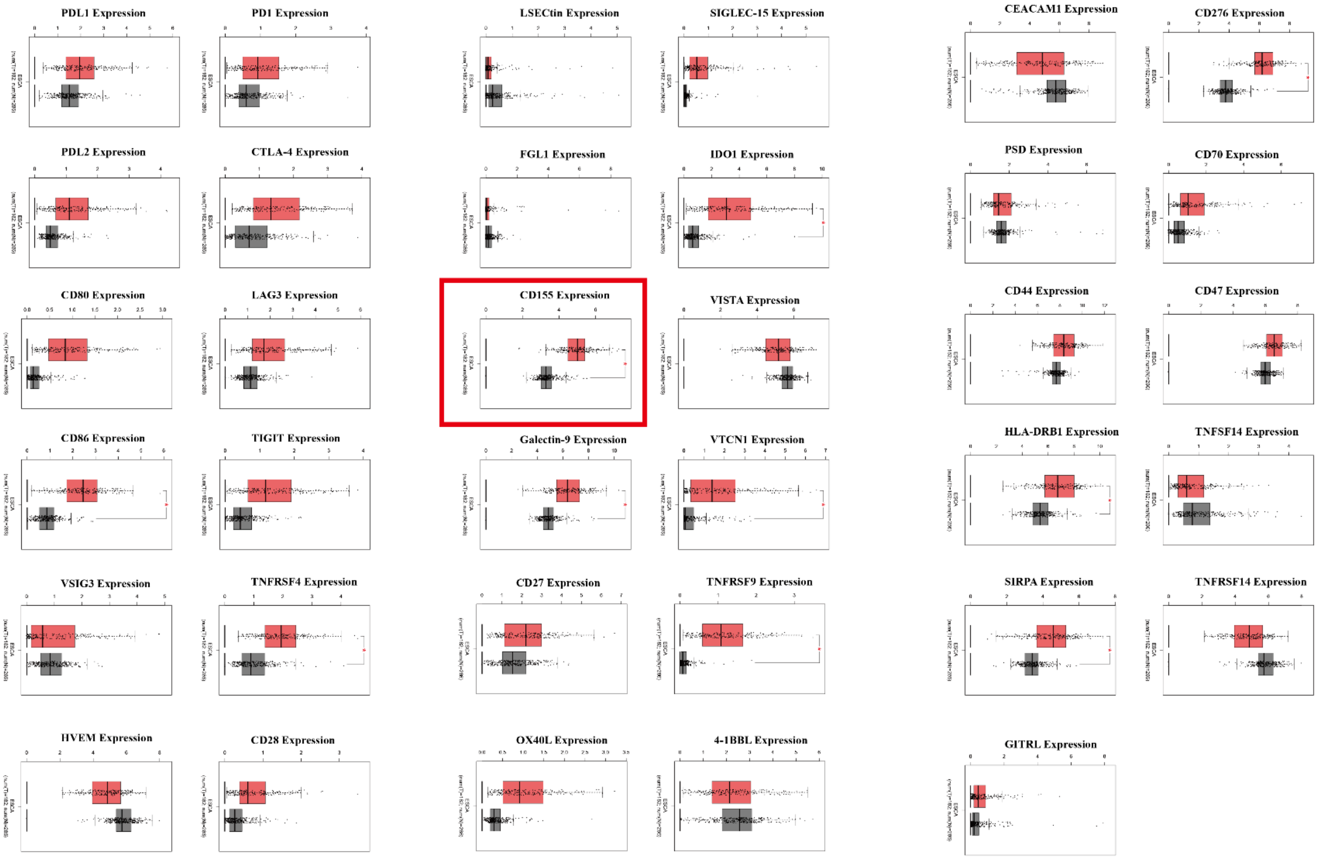

Figure S1. Differential expression of 35 immune checkpoint genes in ESCA. Differential expression of 35 immune checkpoint genes in ESCA were performed in GEPIA database, the differential expression of CD155 is highlighted in red.

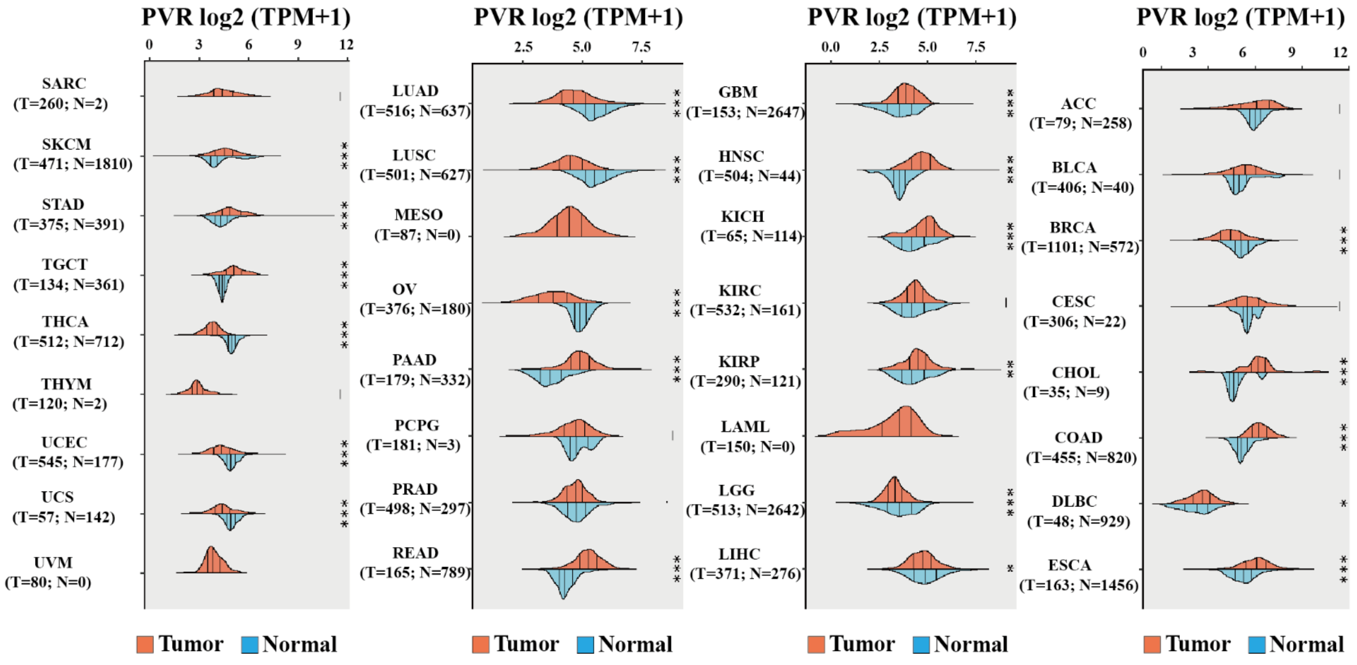

Figure S2. CD155 is also highly expressed in various tumors in addition to ESCA. Pan-cancer analysis from ACLBI database showed higher CD155 expression could also be found in CHOL, COAD and PAAD, as well as other tumors.

A

B

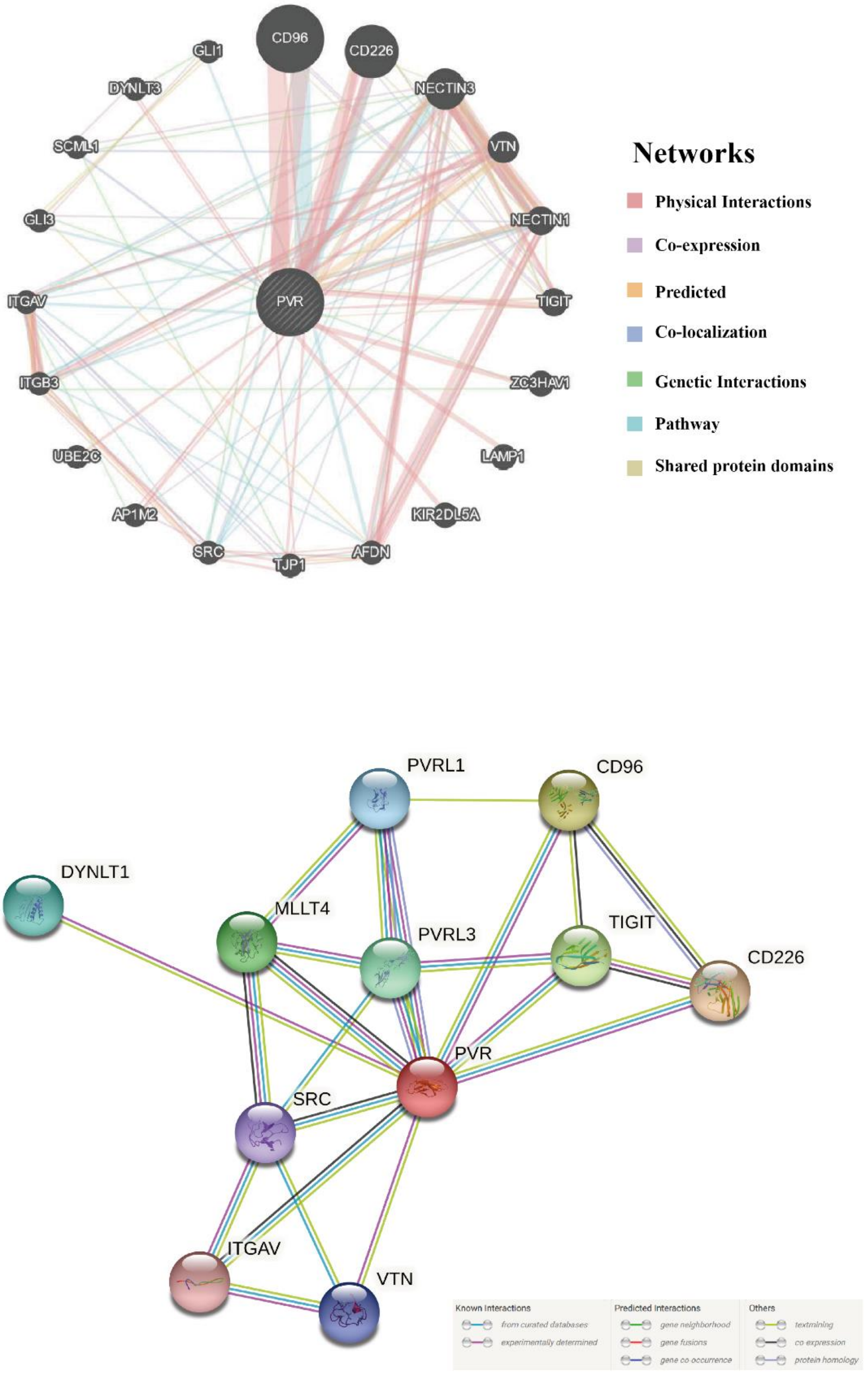

Figure S3. The gene and protein interaction network of CD155. Gene-gene interaction and protein-protein interaction network of CD155 was completed in (**A**) GeneMANIA and (**B**) STRING databases, respectively.

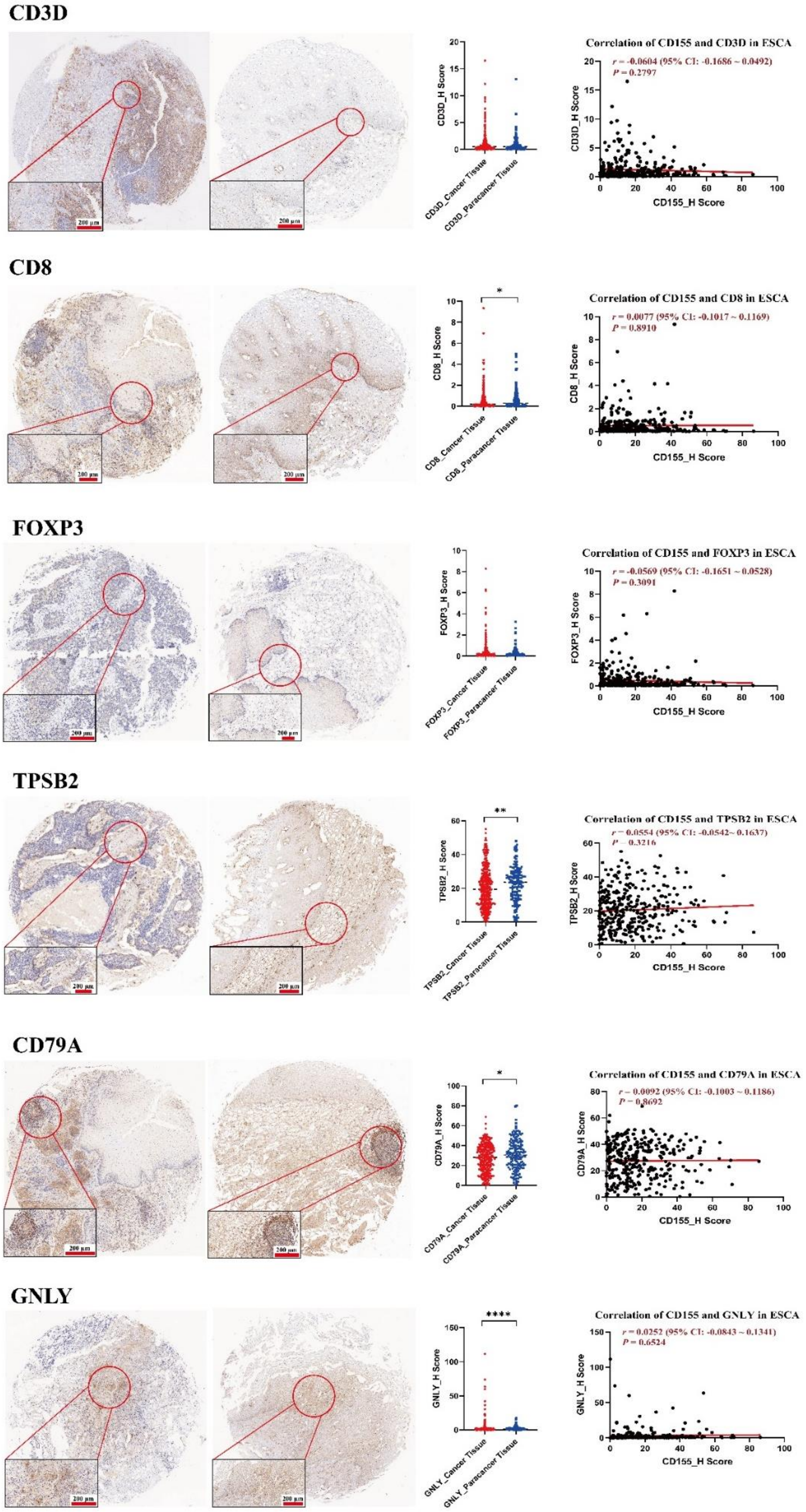

Figure S4. CD155 is not correlated with the expression of various immune markers. 6 immune related markers are stained by immunohistochemistry, correlation analysis between CD155 and immune markers are performed, no any correlations were observed between CD155 and CD3D, CD8, FOXP3, TPSB2, CD79A, GNLY. * $p < 0.05$; ** $p < 0.01$; **** $p < 0.0001$.

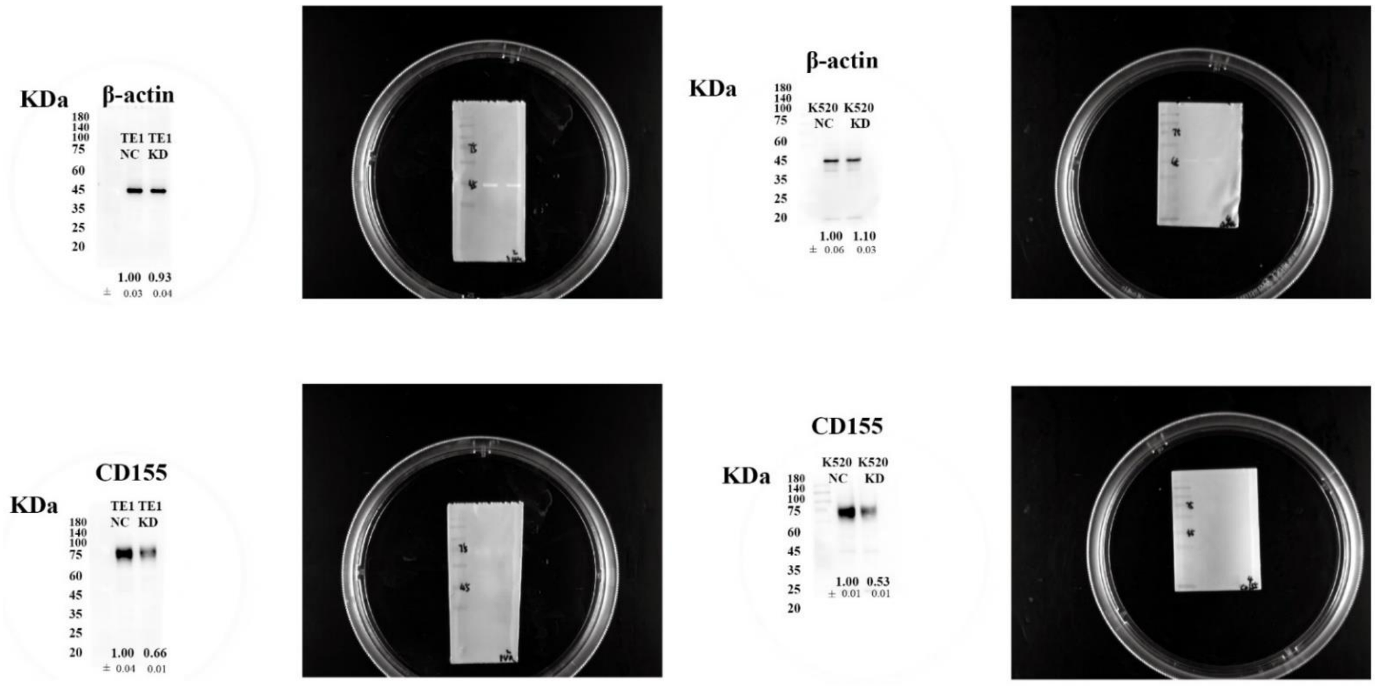

Uncropped WB original images for Figure 7A

Figure S5. The original WB Blots of Figure 7A.

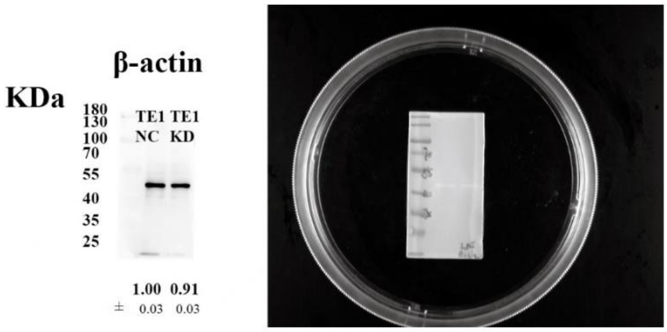
β-actin
KDa
180
130
100
70
55
40
35
25
TE1 NC
TE1 KD
1.00 0.91
± 0.03 0.03

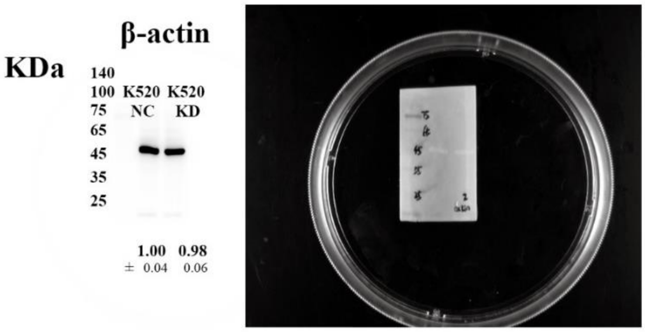
β-actin
KDa
140
100
75
65
45
35
25
K520 NC
K520 KD
1.00 0.98
± 0.04 0.06

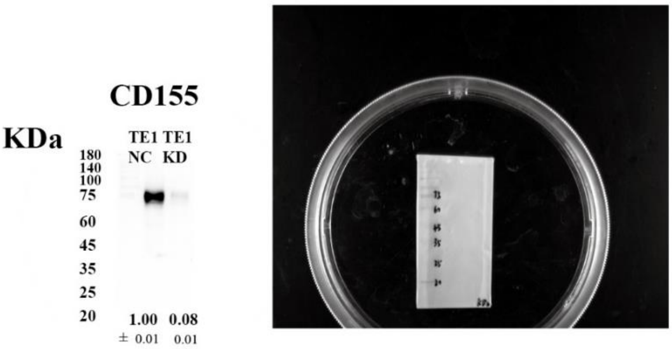
CD155
KDa
180
140
100
75
60
45
35
25
20
TE1 NC
TE1 KD
1.00 0.08
± 0.01 0.01

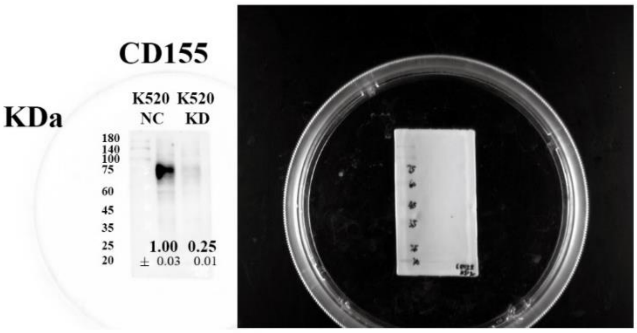
CD155
KDa
180
140
100
75
60
45
35
25
20
K520 NC
K520 KD
1.00 0.25
± 0.03 0.01

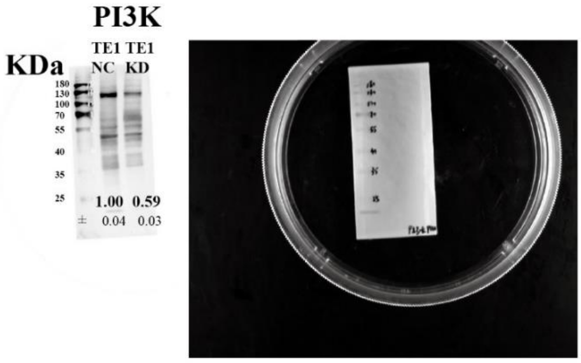
PI3K
KDa
180
130
100
70
55
40
35
25
TE1 NC
TE1 KD
1.00 0.59
± 0.04 0.03

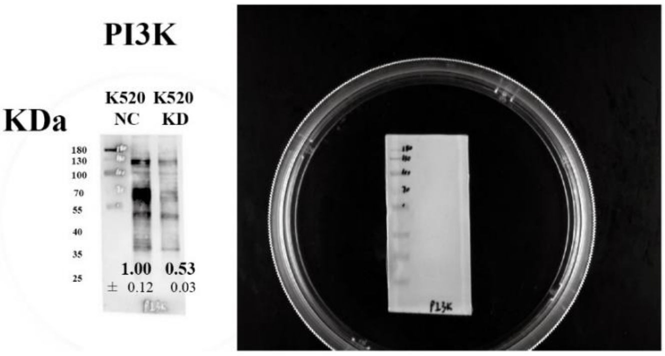
PI3K
KDa
180
130
100
70
55
40
35
25
K520 NC
K520 KD
1.00 0.53
± 0.12 0.03

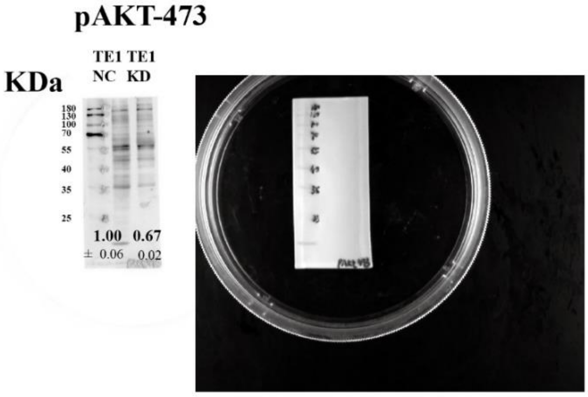
pAKT-473
KDa
180
130
100
70
55
40
35
25
TE1 NC
TE1 KD
1.00 0.67
± 0.06 0.02

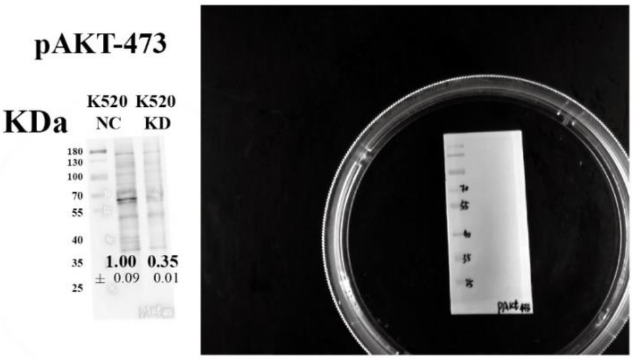
pAKT-473
KDa
180
130
100
70
55
40
35
25
K520 NC
K520 KD
1.00 0.35
± 0.09 0.01

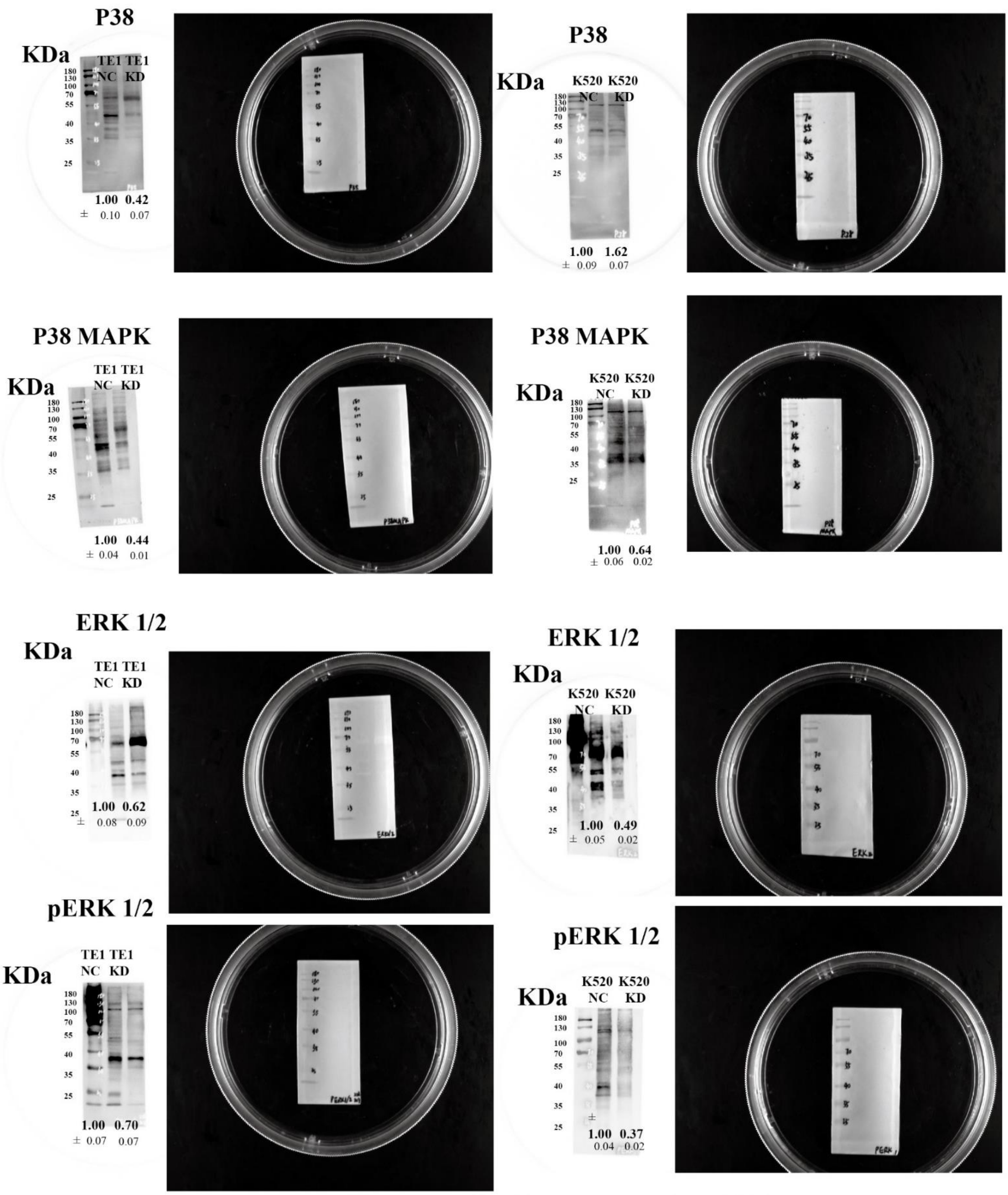
P38
KDa
TE1 NC
TE1 KD
180 130 100 70 55 40 35 25
1.00 0.42
± 0.10 0.07
P38
KDa
K520 NC
K520 KD
180 130 100 70 55 40 35 25
1.00 1.62
± 0.09 0.07
P38 MAPK
KDa
TE1 NC
TE1 KD
180 130 100 70 55 40 35 25
1.00 0.44
± 0.04 0.01
P38 MAPK
KDa
K520 NC
K520 KD
180 130 100 70 55 40 35 25
1.00 0.64
± 0.06 0.02
ERK 1/2
KDa
TE1 NC
TE1 KD
180 130 100 70 55 40 35 25
1.00 0.62
± 0.08 0.09
ERK 1/2
KDa
K520 NC
K520 KD
180 130 100 70 55 40 35 25
1.00 0.49
± 0.05 0.02
pERK 1/2
KDa
TE1 NC
TE1 KD
180 130 100 70 55 40 35 25
1.00 0.70
± 0.07 0.07
pERK 1/2
KDa
K520 NC
K520 KD
180 130 100 70 55 40 35 25
±
1.00 0.37
0.04 0.02

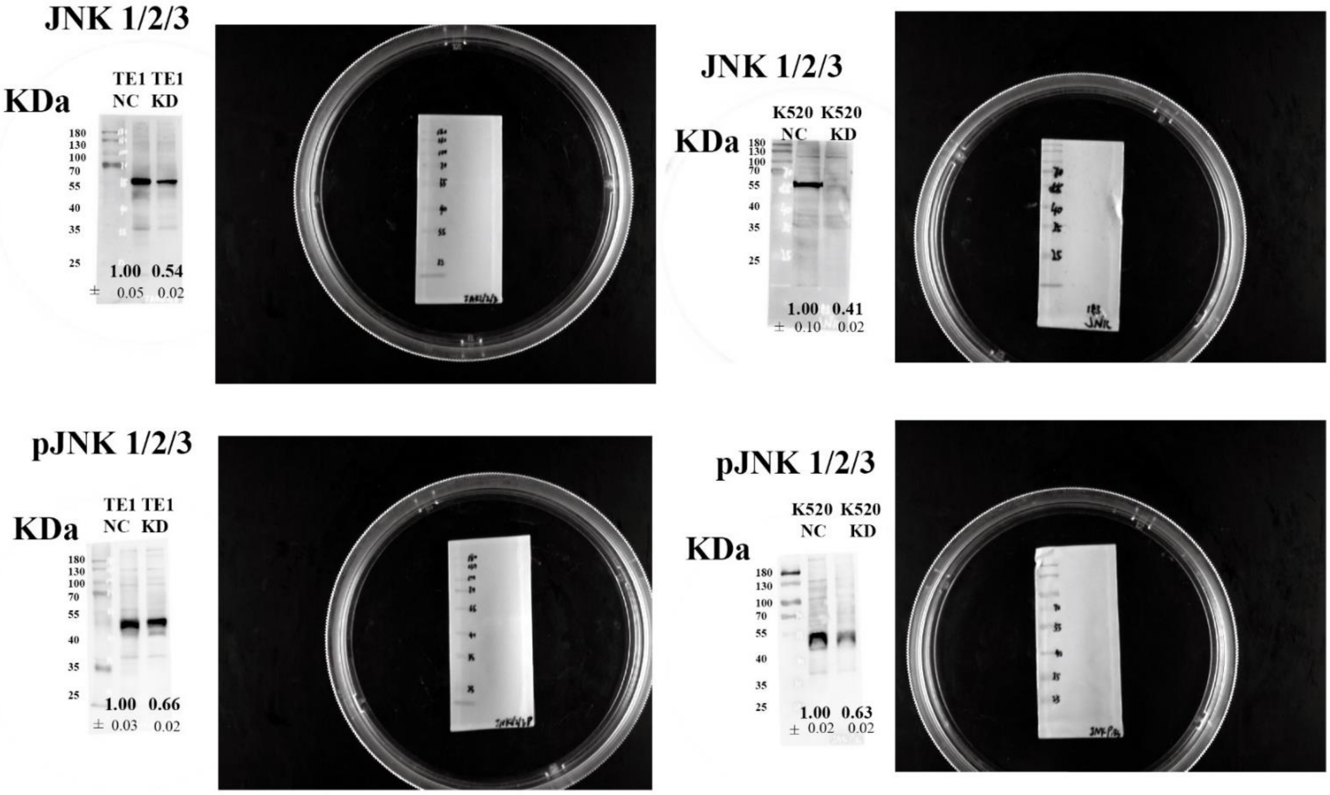

Figure S6. The original WB Blots of Figure 7G.

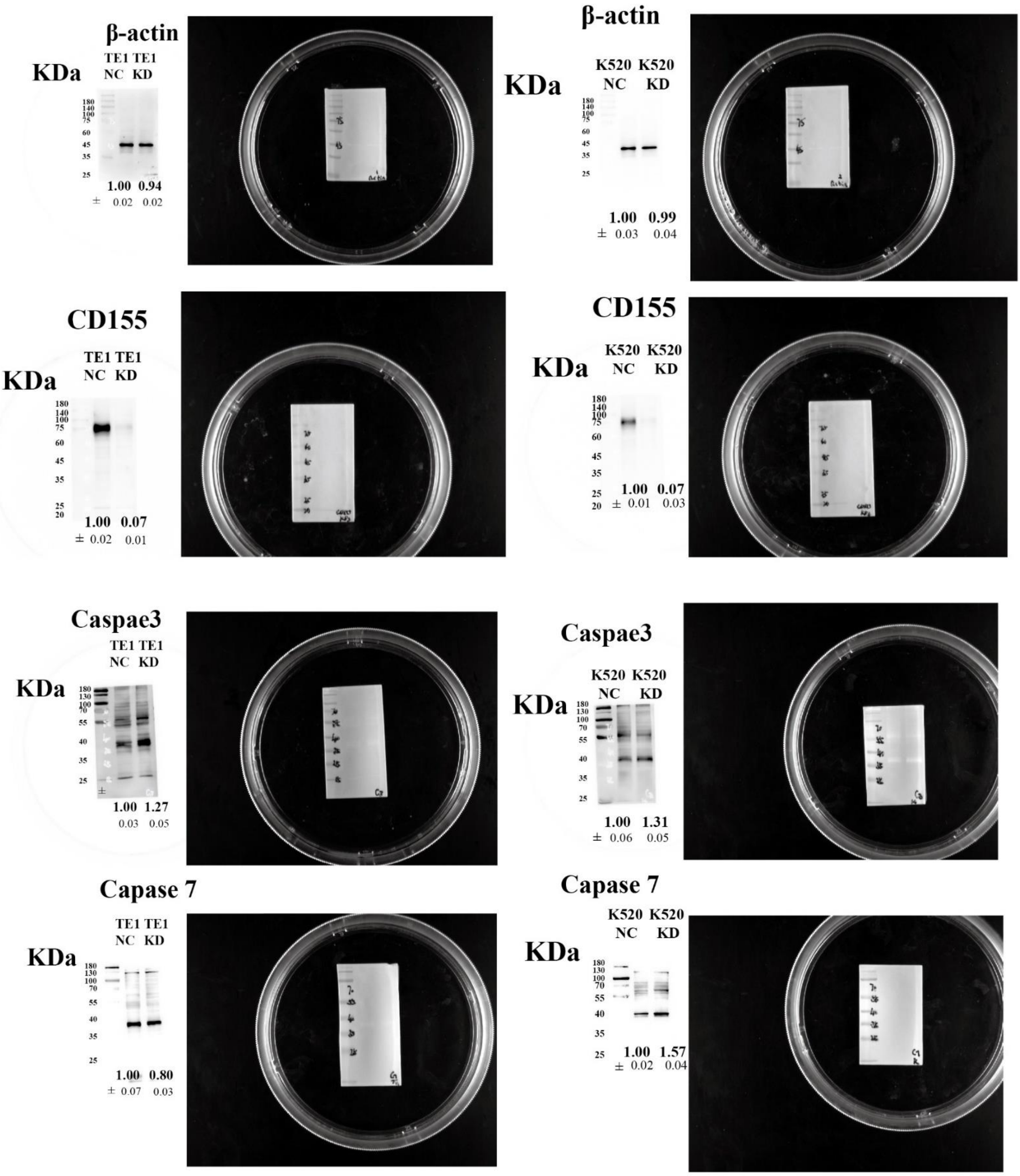
β-actin
KDa
TE1 NC
TE1 KD
1.00 0.94
± 0.02 0.02
β-actin
KDa
K520 NC
K520 KD
1.00 0.99
± 0.03 0.04
CD155
KDa
TE1 NC
TE1 KD
1.00 0.07
± 0.02 0.01
CD155
KDa
K520 NC
K520 KD
1.00 0.07
± 0.01 0.03
Caspae3
KDa
TE1 NC
TE1 KD
1.00 1.27
± 0.03 0.05
Caspae3
KDa
K520 NC
K520 KD
1.00 1.31
± 0.06 0.05
Capase 7
KDa
TE1 NC
TE1 KD
1.00 0.80
± 0.07 0.03
Capase 7
KDa
K520 NC
K520 KD
1.00 1.57
± 0.02 0.04

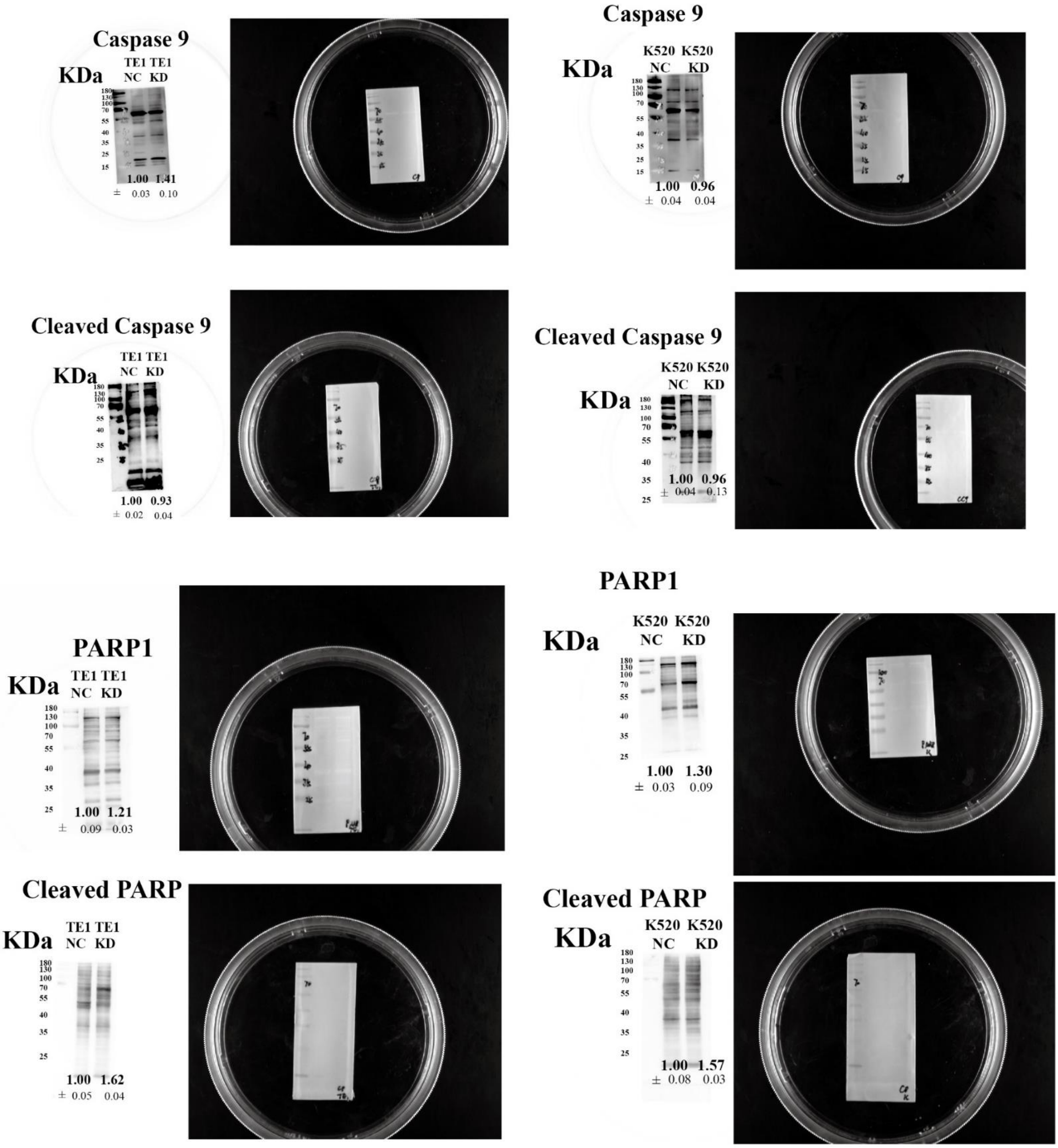

Figure S7. The original WB Blots of Figure 8F.

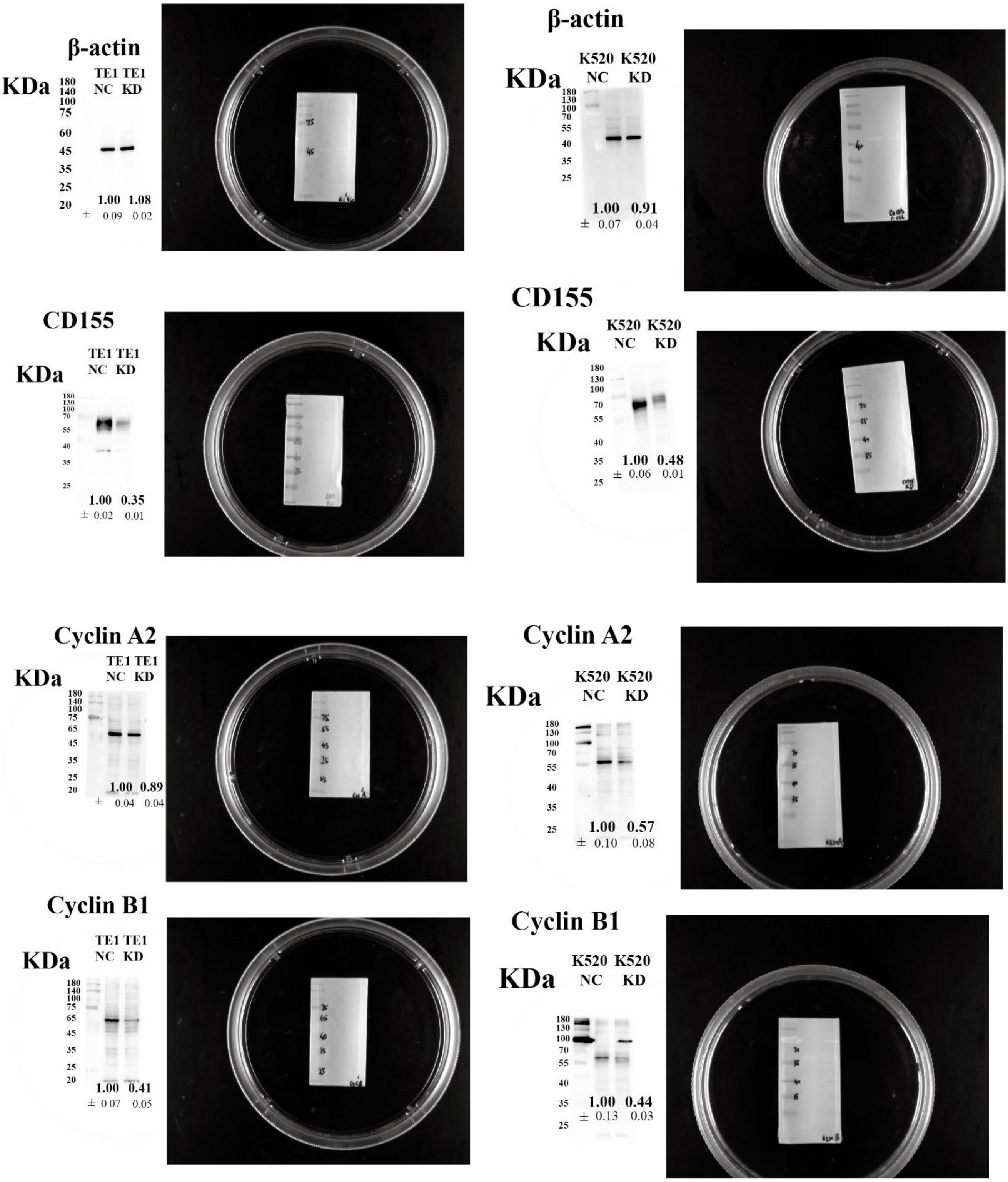
β-actin
KDa
TE1 NC
TE1 KD
180
140
100
75
60
45
35
25
20
1.00
1.08
± 0.09
0.02
β-actin
K520 NC
K520 KD
KDa
180
130
100
70
55
40
35
25
1.00
0.91
± 0.07
0.04
CD155
TE1 NC
TE1 KD
KDa
180
130
100
70
55
40
35
25
1.00
0.35
± 0.02
0.01
CD155
K520 NC
K520 KD
KDa
180
130
100
70
55
40
35
25
1.00
0.48
± 0.06
0.01
Cyclin A2
KDa
TE1 NC
TE1 KD
180
140
100
75
65
45
35
25
20
1.00
0.89
± 0.04
0.04
Cyclin A2
K520 NC
K520 KD
KDa
180
130
100
70
55
40
35
25
1.00
0.57
± 0.10
0.08
Cyclin B1
TE1 NC
TE1 KD
KDa
180
140
100
75
65
45
35
25
20
1.00
0.41
± 0.07
0.05
Cyclin B1
K520 NC
K520 KD
KDa
180
130
100
70
55
40
35
25
1.00
0.44
± 0.13
0.03

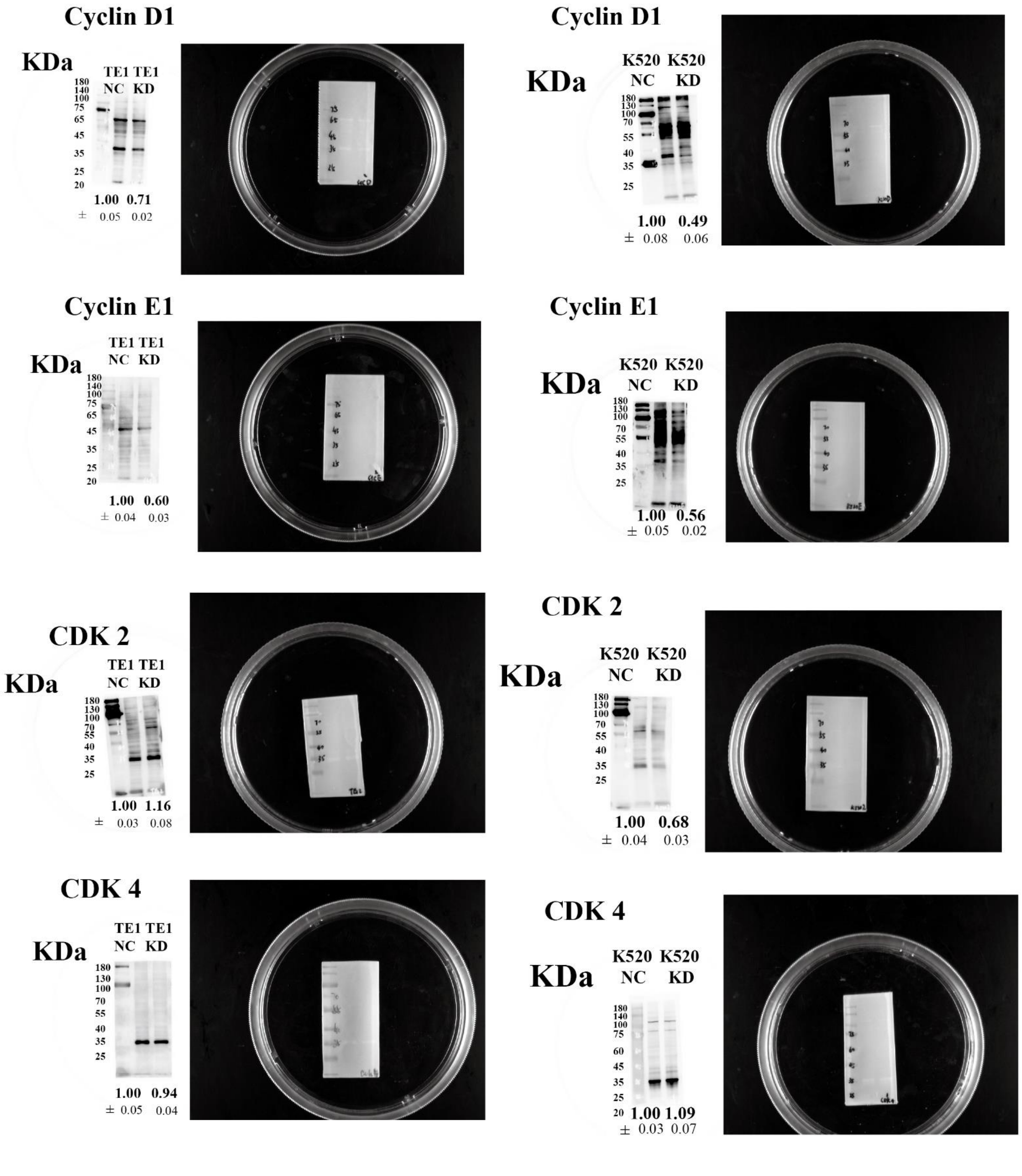
Cyclin D1
KDa
TE1 NC
TE1 KD
180
140
100
75
65
45
35
25
20
1.00 0.71
± 0.05 0.02
Cyclin D1
KDa
K520 NC
K520 KD
180
130
100
70
55
40
35
25
1.00 0.49
± 0.08 0.06
Cyclin E1
KDa
TE1 NC
TE1 KD
180
140
100
75
65
45
35
25
20
1.00 0.60
± 0.04 0.03
Cyclin E1
KDa
K520 NC
K520 KD
180
130
100
70
55
40
35
25
1.00 0.56
± 0.05 0.02
CDK 2
KDa
TE1 NC
TE1 KD
180
130
100
70
55
40
35
25
1.00 1.16
± 0.03 0.08
CDK 2
KDa
K520 NC
K520 KD
180
130
100
70
55
40
35
25
1.00 0.68
± 0.04 0.03
CDK 4
KDa
TE1 NC
TE1 KD
180
130
100
70
55
40
35
25
1.00 0.94
± 0.05 0.04
CDK 4
KDa
K520 NC
K520 KD
180
140
100
75
60
45
35
25
20
1.00 1.09
± 0.03 0.07

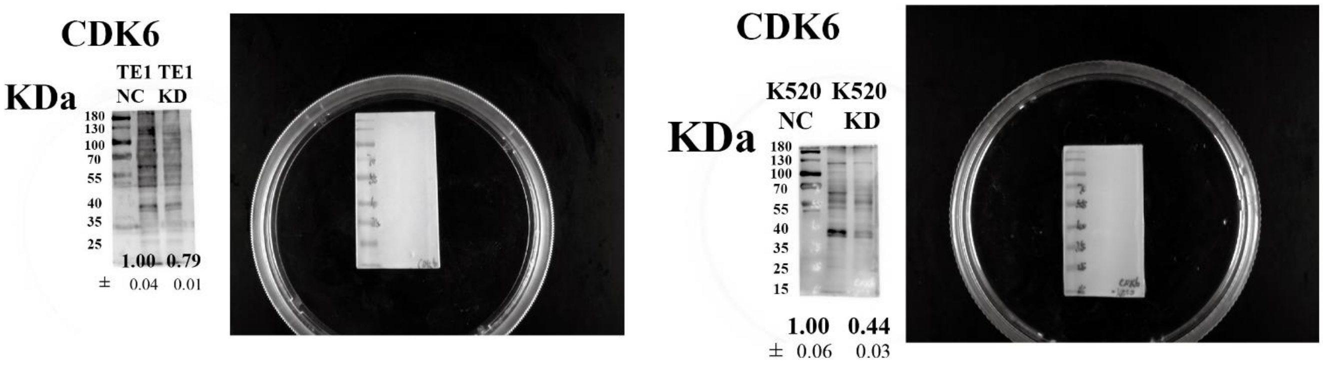

Figure S8. The original WB Blots of Figure 8G.

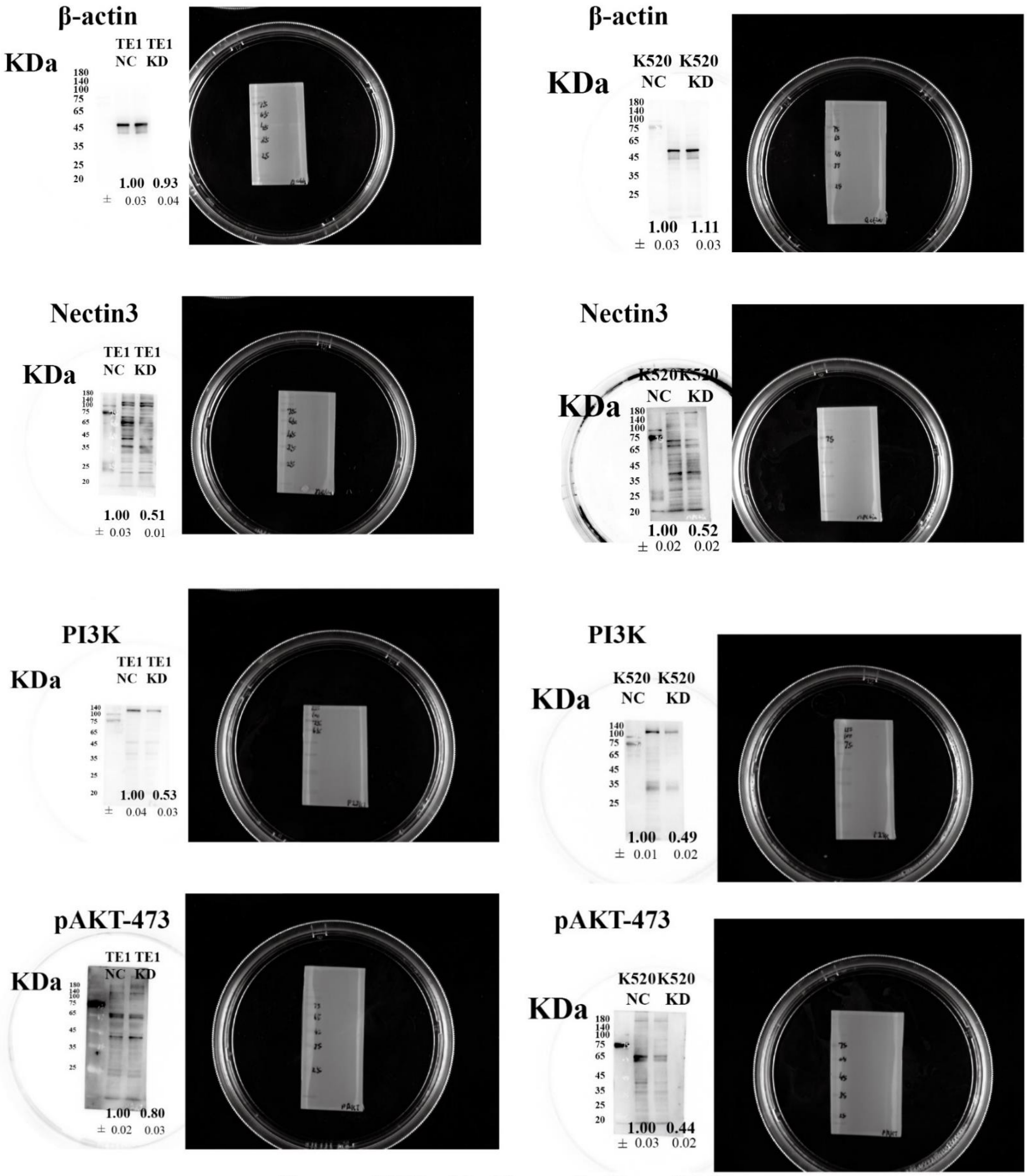
β-actin
KDa
TE1 NC
TE1 KD
180
140
100
75
65
45
35
25
20
1.00 ± 0.03
0.93 0.04
β-actin
KDa
K520 NC
K520 KD
180
140
100
75
65
45
35
25
1.00 ± 0.03
1.11 0.03
Nectin3
KDa
TE1 NC
TE1 KD
1.00 ± 0.03
0.51 0.01
Nectin3
KDa
K520 NC
K520 KD
180
140
100
75
65
45
35
25
20
1.00 ± 0.02
0.52 0.02
PI3K
KDa
TE1 NC
TE1 KD
140
100
75
65
45
35
25
20
1.00 ± 0.04
0.53 0.03
PI3K
KDa
K520 NC
K520 KD
140
100
75
65
45
35
25
1.00 ± 0.01
0.49 0.02
pAKT-473
KDa
TE1 NC
TE1 KD
1.00 ± 0.02
0.80 0.03
pAKT-473
KDa
K520 NC
K520 KD
180
140
100
75
65
45
35
25
20
1.00 ± 0.03
0.44 0.02

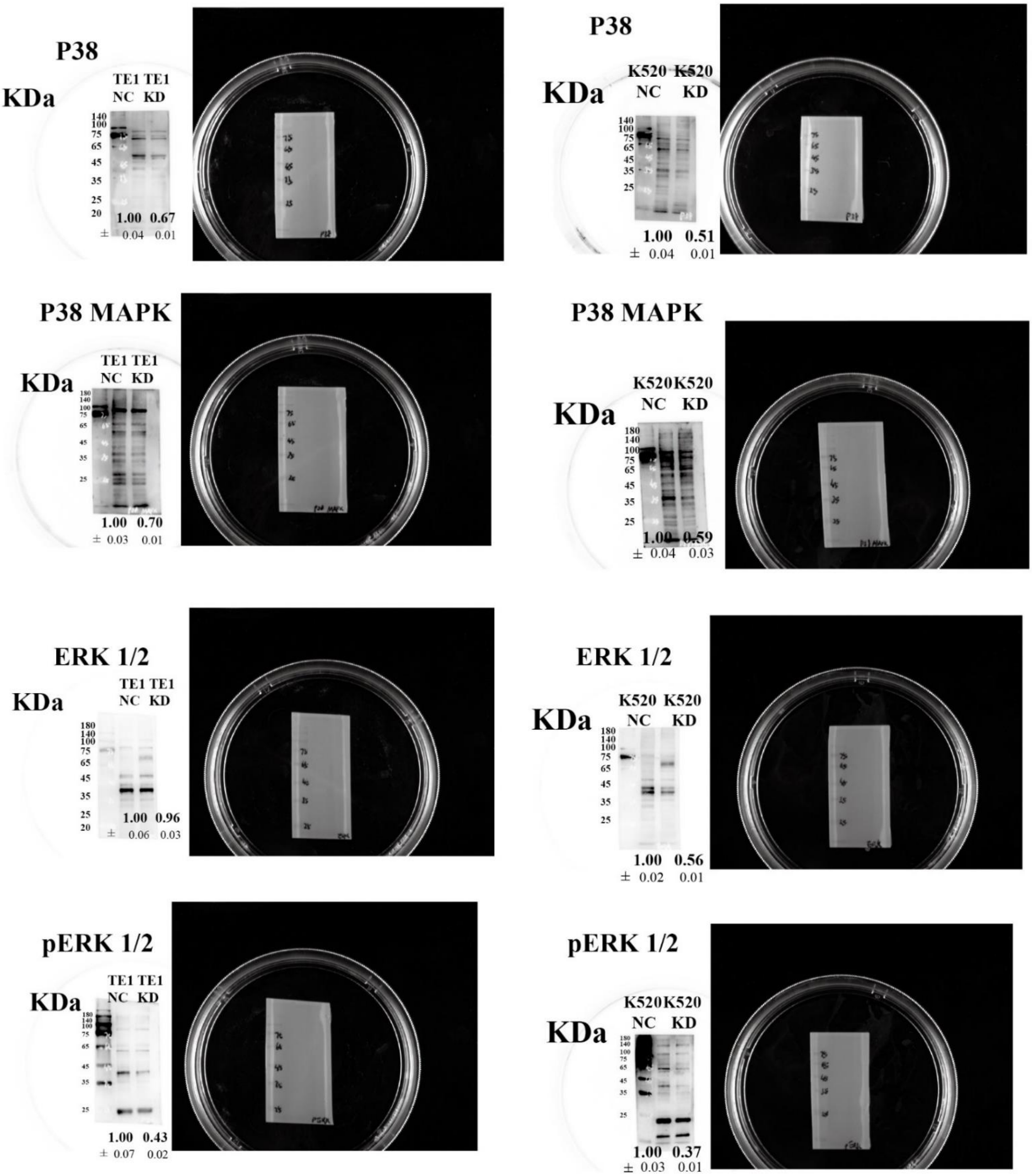

P38
KDa
TE1 NC
TE1 KD
140
100
75
65
45
35
25
20
1.00 0.67
± 0.04 0.01
P38
KDa
K520 NC
K520 KD
140
100
75
65
45
35
25
1.00 0.51
± 0.04 0.01
P38 MAPK
KDa
TE1 NC
TE1 KD
1.00 0.70
± 0.03 0.01
P38 MAPK
KDa
K520 NC
K520 KD
180
140
100
75
65
45
35
25
1.00 0.59
± 0.04 0.03
ERK 1/2
KDa
TE1 NC
TE1 KD
180
140
100
75
65
45
35
25
20
1.00 0.96
± 0.06 0.03
ERK 1/2
KDa
K520 NC
K520 KD
180
140
100
75
65
45
35
25
1.00 0.56
± 0.02 0.01
pERK 1/2
KDa
TE1 NC
TE1 KD
1.00 0.43
± 0.07 0.02
pERK 1/2
KDa
K520 NC
K520 KD
1.00 0.37
± 0.03 0.01

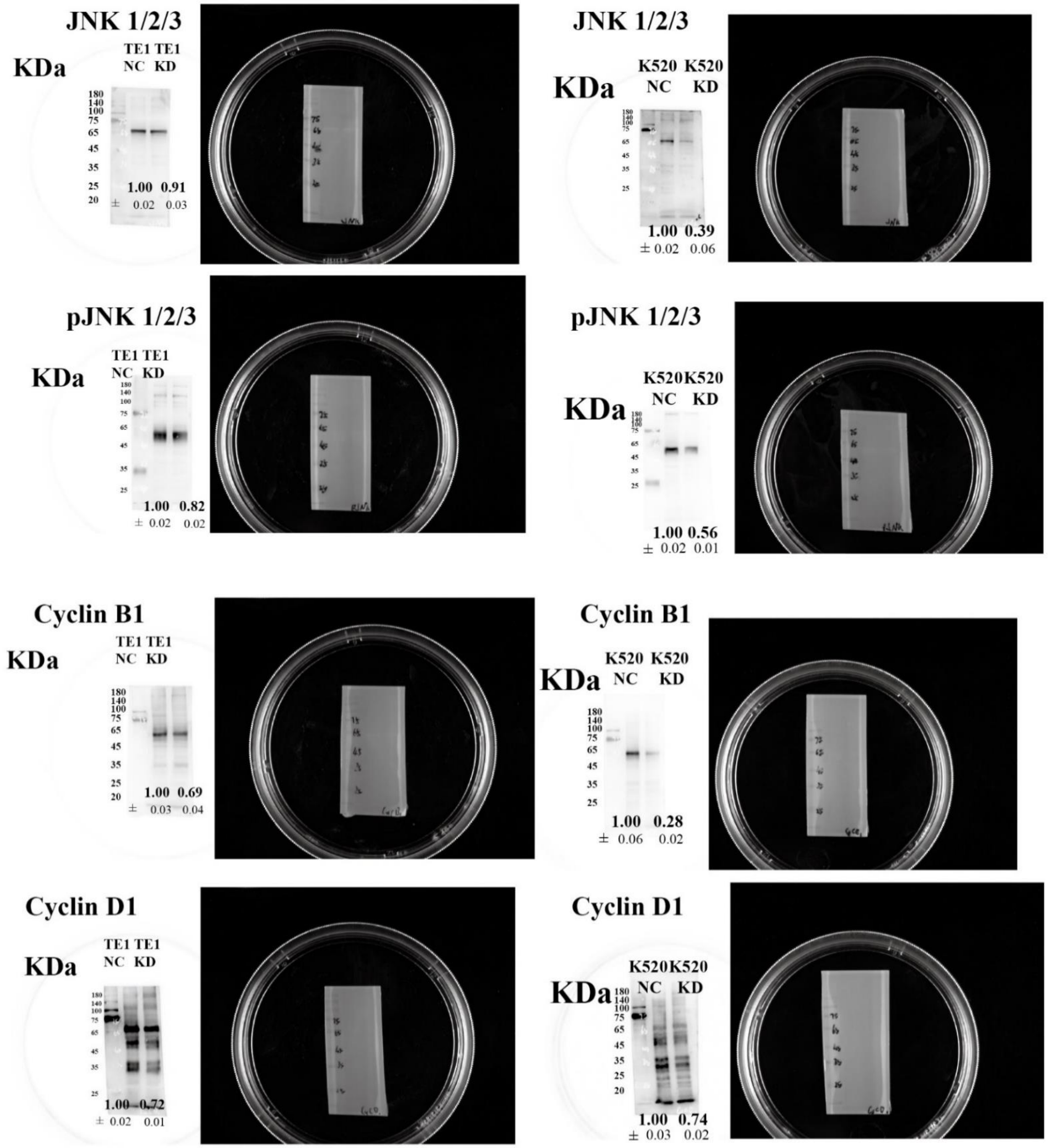
JNK 1/2/3
KDa
TE1 NC
TE1 KD
180
140
100
75
65
45
35
25
20
1.00 0.91
± 0.02 0.03
JNK 1/2/3
KDa
K520 NC
K520 KD
1.00 0.39
± 0.02 0.06
pJNK 1/2/3
KDa
TE1 NC
TE1 KD
1.00 0.82
± 0.02 0.02
pJNK 1/2/3
KDa
K520 NC
K520 KD
1.00 0.56
± 0.02 0.01
Cyclin B1
KDa
TE1 NC
TE1 KD
1.00 0.69
± 0.03 0.04
Cyclin B1
KDa
K520 NC
K520 KD
1.00 0.28
± 0.06 0.02
Cyclin D1
KDa
TE1 NC
TE1 KD
1.00 0.72
± 0.02 0.01
Cyclin D1
KDa
K520 NC
K520 KD
1.00 0.74
± 0.03 0.02

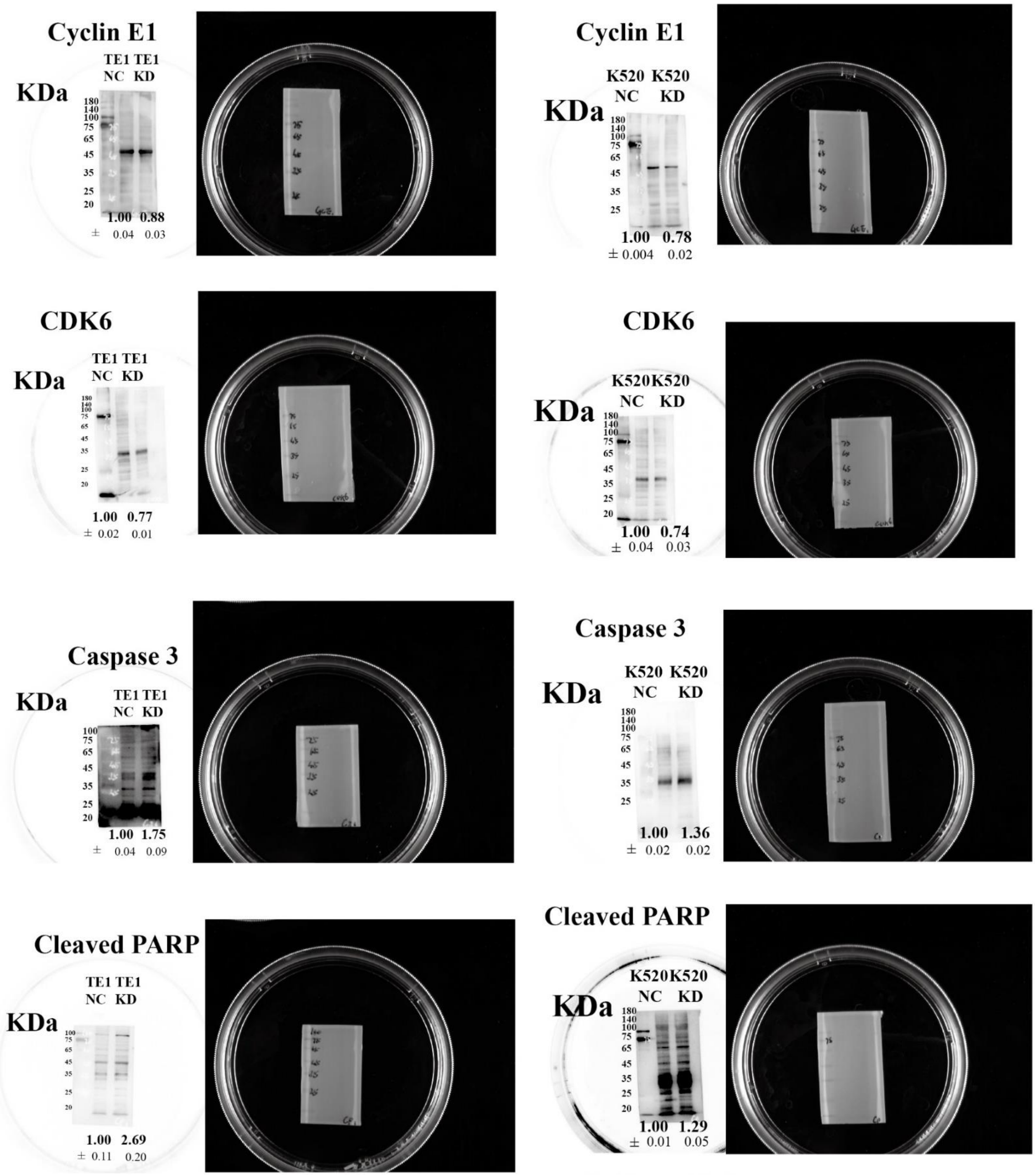

Figure S9. The original WB Blots of Figure 9C.
